# Modification of an *Anopheles gambiae* odorant binding protein to create an array of chemical sensors for detection of drugs

**DOI:** 10.1038/s41598-020-60824-7

**Published:** 2020-03-03

**Authors:** Khasim Cali, Krishna C. Persaud

**Affiliations:** Department of Chemical Engineering and Analytical Science, The University of Manchester, Manchester, M13 9PL UK

**Keywords:** Biomimetics, Protein design

## Abstract

The binding pockets of odorant binding proteins from *Anopheles gambiae (OBP1* and *OBP47)* were analysed using *in silico* modelling. The feasibility of creating mutant proteins to achieve a protein array capable of detecting drugs of abuse in solution or in vapour phase was investigated. OBP1 was found to be easily adapted and several mutant proteins were expressed and characterised. AgamOBP1_S82P was found to have high affinities to cannabinol, 3,4-methylenedioxy methamphetamine (MDMA/Ecstasy) and cocaine hydrochloride. When these proteins were immobilised on a quartz crystal microbalance, saturated cocaine hydrochloride vapour could be detected. The sensors were stable over a period of at least 10 months in air. The approach taken allows flexible design of new biosensors based on inherently stable protein scaffolds taking advantage of the tertiary structure of odorant binding proteins.

## Introduction

In vertebrates and insects, the process of chemoreception involves transmembrane receptors (olfactory receptors (ORs)) that are responsible for the transduction of a signal associated with the binding of a ligand to a receptor binding site^1,2^. However many volatile molecules of interest are generally hydrophobic and before reaching the dendrites of sensory neurons, they need to partition from air into an aqueous environment before they can reach the receptors^3^. The nasal mucus of non-aquatic vertebrates and the chemosensillar lymph of insect antennae contain large amounts of small soluble odorant-binding proteins (OBPs) that specifically and reversibly bind odour molecules and pheromones. In vertebrates these proteins form part of the lipocalin family – low molecular weight proteins characterised by a cage-like structure of beta-sheets that function as carriers of small ligands^4^. In insects there are two major families - OBPs and chemosensory proteins (CSPs), with a sub-group of OBPs, specifically tuned to pheromones, referred to as PBPs^5,6^. These proteins are also of low molecular weight but primarily consisting of alpha-helical structures in contrast to those of vertebrates.

While it would be desirable to use olfactory receptors directly as biosensor recognition elements, there are practical difficulties because ORs are membrane bound G protein-coupled receptors (GPCRs)^1^ or olfactory receptor-co-receptors (ORCO)^7,8^ and it is much more difficult to stabilise these proteins outside the cellular environment. On the other hand, OBPs are attractive for use as bio-recognition elements for small ligands and have attracted the attention of a number of researchers who have successfully demonstrated that they can function as biosensors^9–11^. Mulla *et al*. demonstrated that the two enantiomers of carvone could be distinguished using a water gated field effect transistor^12^. Larisika *et al*. immobilised OBP14 from the honeybee on graphene and incorporated it into a field-effect transistor to produce biosensors able to discriminate ligands in a way that was similar to the specificity of the protein when measured in solution^13^. These developments are due to the large body of information now available on structure and function of OBPs in a variety of species. They can be easily synthesised in bulk in heterologous expression systems, due to their small size and lack of post-translational modifications. They are resistant to degradation by proteolysis, stable over a large temperature range, and can withstand organic solvents^14^. The native proteins bind a large variety of small ligands and while selectivity is rather broadly tuned, individual proteins are unique in the range of molecules that they can interact with. There is great interest in detection, screening and identifying drugs of abuse in several scenarios – e.g. detection of contraband drugs smuggled across borders^15^ and importantly by harm-reduction agencies and clinics who seek to decrease the risk of adverse effects in drug users, where often it is not known what substances have been consumed. While canines represent the state of the art for sniffing out drugs and explosives^16^ the best instrumental methods available for drug testing currently are handheld infrared spectroscopy, Raman spectroscopy, and ion mobility spectrometry; with mass spectrometry being the current gold standard in forensic drug analysis. For rapid screening and identification of a drug the most commonly used tool is ion mobility spectrometry (IMS), where a swab of an object is taken and thermally desorbed into the detector^17^. IMS is highly attractive for field use because it can be used by non-technical personnel and is routinely used for detecting trace levels of illicit drugs. However, it has not been used for the identification of bulk drug samples because of problems of saturation, memory effects and inability to discriminate the presence of a drug within complex mixtures^18^. Current instrumental methods generally suffer from a lack of efficient sampling systems, selectivity problems in the presence of interfering odour chemicals and limited mobility/tracking ability^16^. Hence better detection methods are being sought, one approach being to utilise biorecognition elements taken directly from biological chemoreception systems.

The problem we address here is the design of proteins that can be tailored to bind drugs of abuse to create arrays of biosensors that can improve on the current tests available^19^. Ligands of interest include cocaine, ephedrine, tetrahydro cannabinol (THC), 3,4-methylenedioxy methamphetamine (MDMA/Ecstasy) and heroin. We focus on two proteins from *Anopheles gambiae* – OBP1 and OBP47 that have widely different ligand binding behaviours to ligands in solution^20^. OBP1 is expressed in the antenna of the insect, while OBP47 is expressed in the head without antennae – mouth structures such as the palpi and proboscis, with a suggested putative function in taste^21^. It has been shown that silencing of the gene encoding OBP1 in *A. gambiae*^22^ and in *Culex quinquefasciatus*^23^ suppresses electrophysiological responses to indole, indicating that OBP1 may be involved in the perception of indole.

Insect OBPs exhibit great similarities in structure, with a six α-helix core, an internal cavity and three disulphide bridges, features that define them as classical OBPs. However there are significant differences at their C-terminal, with functional implications^24^ and these classical OBPs are divided into long, medium and short-C terminal subclasses^25^. AgamOBP1 belongs to the medium subclass, possessing an elongated C-terminal segment buried inside the protein core, forming a wall with the internal cavity; it is 125 residues long and has six cysteines with three disulphide bonds. OBPs are also classified into distinct other classes, where those with four cysteines are called C-minus class^26^, OBPs with a longer chain and 12 or more cysteines, the C-plus class, together with OBPs with double domains of classical OBPs, called double OBPs^27^. Unlike AgamOBP1, AgamOBP47 belongs to the C-plus OBP class, is 173 residues long, possesses 13 cysteine residues with six disulphide bonds and its structure is mostly helical, being similar with classical OBPs. However, eight α-helices are observed in OBP47 rather than the six in classical OBPs such as AgamOBP1.

The design of mutant proteins targeting protein functional regions, such as the ligand binding sites, is a powerful approach often used to recognise the determinants of specific protein activities in cellular pathways. Large scale mutagenesis techniques are often employed for an exhaustive analysis of selected positions of protein structure, but this is laborious and time consuming. *In silico* mutagenesis and screening simulation can be a valid alternative to laboratory methods to drive the ‘*in vivo*’ testing toward more focused objectives. The procedure involves a saturation mutagenesis of all residues involved in ligand binding with subsequent evaluation of the effect of amino acid substitutions on ligand affinity by docking simulation and on protein structure stability followed by a rationally driven selection of those presenting the required characteristics^28^. Here we show how site directed substitutions of single amino acid residues in OBP binding pockets allow tailoring of the protein to bind non-native ligands, resulting in the development of an array of odorant binding proteins for detection of volatile and semi-volatile molecules. OBP1 was compared with OBP47 in terms of potential for creation of stable mutants that could bind the ligands of interest. OBP1 had much better potential for creation of suitable mutants and was selected as a template for creating a range of modified OBPs which display different specificities to different drugs of interest.

## Results

### Characterisation of the Anopheles gambiae OBP1 and OBP 47 binding pockets

The creation of stable mutant proteins capable of binding the ligands of interest were investigated.

The X-ray structure (PBD accession number: 2ERB) of *Anopheles gambiae* OBP1 (AgamOBP1) complexed with polyethylene glycol (PEG) was used as a starting point **(**Supplementary Fig. S1**)**. This was chosen against other available AgamOBP1- ligand complex structures such as AgamOBP1- DEET (PDB ID: 3N7H), Icaridin (PDB ID: 5EL2), and 6-MH (6-methyl-5-heptene-2-one) (PDB ID: 4FQT) because PEG is a larger molecule compared to the other ligands spanning the whole of the AgamOBP1 monomer through the entire tunnel shaped active site of the protein^29–31^ (Supplementary Fig. S2). The LPC/CSU (Weizmann, AC) server was used to identify binding pocket (active site) residues that interact with the ligands in AgamOBP1, based on the X-ray structure of the protein. The available X-ray structure of AgamOBP47 (PDB ID: 3PM2) was not solved as a complex with a ligand^32^, so the LPC/CSU (Weizmann, AC) server could not be used for this protein. The CASTp server was used instead to identify all potential binding pockets within the protein **(**Supplementary Fig. S3). For comparison all potential binding pockets in AgamOBP1 were also identified using CASTp. Following LPC/CSU analysis, for AgamOBP1, 31 amino acids were identified to have direct contact with the ligand (Table 1). Of these, 23 (74%) were hydrophobic in nature. *In silico* analysis of the binding pocket residues (analysing potential mutant stability using rigidity analysis (KINARI MUTAGEN SERVER)^33^ identified that 13 (residues highlighted in bold in Table 1) of these 31 residues were feasible for mutations without compromising the binding pocket or whole protein integrity. Energy analysis via direct amino acid substitutions identified 28 possibly stable mutants (Supplementary Table S1) at 6 different binding pocket positions (PoPMuSiC Program^34^). Of these, eighteen (64.3%) were when the new residues were hydrophobic, four (14.2%) mutants when the new residues were positive, only one (3.6%) mutant when the new residues were negative and the remaining five (17.9%) mutants were when the new residues were hydrophilic (Table 2). Table 1 shows that the six residues that gave the stable mutants generally have greater distances to the corresponding atom of the ligand compared to all other residues.

**Table 1:** The LPC/CSU (Weizman, AC) server was used to identify binding pocket (active site) residues that interact with the PEG ligand in AgamOBP1_PEG complex X-ray structure (PDB 2ERB).

Residue	Dist	Surf	HB	Ar	Ph	D	Rn
14 GLU*	3.6	41.0	—	—	—	+	Ng
15 LEU*	3.9	47.1	—	—	—	—	Hb
18 ALA*	3.5	29.2	—	—	—	+	Hb
19 LEU*	3.8	42.8	—	—	—	—	Hb
22 LEU*	3.8	32.1	—	—	—	—	Hb
58 LEU*	3.5	58.0	—	—	—	+	Hb
59 PHE*	**3.8**	**18.4**	**—**	**—**	**—**	**—**	**Hb**
62 ALA*	3.4	22.2	—	—	—	+	Hb
64 VAL*	**4.7**	**1.6**	**—**	**—**	**—**	**—**	**Hb**
73 LEU*	3.9	36.1	—	—	—	+	Hb
76 LEU*	**3.8**	**48.9**	**—**	**—**	**—**	**—**	**Hb**
77 HIS*	**3.8**	**31.0**	**—**	**—**	**—**	**+**	**Pt**
**78 ASP**	**4.4**	**5.2**	**+**	**—**	**—**	**—**	**Ng**
79 SER*	**3.0**	**70.7**	**+**	**—**	**—**	**+**	**Hp**
80 LEU*	**3.7**	**45.0**	**+**	**—**	**—**	**—**	**Hb**
81 PRO*	**3.9**	**36.0**	**—**	**—**	**—**	**+**	**Hb**
82 SER	5.5	0.2	—	—	—	—	Hp
84 MET*	3.5	22.5	—	—	—	+	Hb
88 ALA*	3.5	45.6	—	—	—	+	Hb
89 MET*	3.8	23.1	—	—	—	+	Hb
91 MET*	3.3	37.5	—	—	—	+	Hb
92 GLY*	4.3	17.7	—	—	—	+	Hb
93 LYS*	**4.2**	**14.2**	**—**	**—**	**—**	**+**	**Pt**
96 LEU*	3.8	37.9	—	—	—	+	Hb
111 HIS*	**4.4**	**17.2**	**+**	**—**	**—**	**—**	**Pt**
114 TRP*	**3.8**	**36.8**	**+**	**—**	**—**	**—**	**Hb**
121 HIS	4.8	0.4	—	—	—	+	Pt
122 TYR*	**4.0**	**17.5**	**—**	**—**	**—**	**—**	**Hb**
123 PHE*	**3.6**	**47.6**	**—**	**—**	**—**	**+**	**Hb**
124 LEU*	4.9	4.9	—	—	—	—	Hb
125 VAL*	6.0	1.3	—	—	—	—	Hb
Total = 31							

Amino acids involved in the binding pocket of AgamOBP1 are listed, showing the properties of each specific contact. **Dist**: nearest distance (Å) between atoms of the ligand and the residue, **Surf**: contact surface area (Å^2^) between the ligand and the residue, **HB**: hydrophilic‐hydrophilic contact (hydrogen bond), **Arom**: aromatic‐aromatic contact, **Phob**: hydrophobic-hydrophobic contact, **DC**: hydrophobic‐hydrophilic contact (destabilizing contact, +/− indicates presence/absence of a specific contact, * indicates residues contacting ligand by their side chain). **Rn**: Residue nature, **Hb**: Hydrophobic, **Hp**: Hydrophilic, **Pt**: Positive, **Ng**: Negative. Residues highlighted in **bold** were identified as feasible for mutation.

**Table 2:** Binding Pocket Features – AgamOBP1 and AgamOBP47.

Main binding pocket (Active site)	AgamOBP1	AgamOBP47
Area (Solvent accessible surface - AS) Å^2^	288.917	765.38
Area (Molecular surface - MS) Å^2^	597.09	1318.58
Volume (Solvent accessible surface - AS) Å^3^	170.011	664.812
Volume (Molecular surface - MS) Å^3^	768.1	2050.87
Pocket length (Å)	329.18	651.82
**Number of mouth openings**	**2**	**3**
Mouth area (Solvent accessible surface - AS) Å^2^	8.46	150.314
Mouth area (Molecular surface - MS) Å^2^	57.96	366.42
Mouth Length (Solvent accessible surface - AS) Å	26.47	143.308
Mouth Length (Molecular surface - MS) Å	44.93	168.99
**Number of binding pocket residues/whole protein**	31/125, (25%)	60/173, (35%)
Binding pocket residues - Hydrophobic	23(74%)	39 (65%)
Binding pocket residues - Hydrophilic	2 (6.5%)	13 (21.6%)
Binding pocket residues - Positive	4 (13%)	5(8.3%)
Binding pocket residues - Negative	2 (6.5%)	3 (5%)
**Feasible positions to mutate (KINARI MUTAGEN)**	**13/31, (42%)**	**27/60, (45%)**
**Number of residues that gave stable mutants (PoPMuSiC)**	**6/31, (19%)**	**18/60, (30%)**
Residues that gave stable mutants - Hydrophobic	2 (33.3%)	7 (39.9%)
Residues that gave stable mutants - Hydrophilic	1(16.6%)	5 (27.8%)
Residues that gave stable mutants - Positive	2 (33.3%)	3 (16.7)
Residues that gave stable mutants - Negative	1 (16.0%)	3 (16.7%)
**Total number of stable mutants**	**28**	**79**
Stable mutants - when a new residue is Hydrophobic	18 (64.3)	49 (62%)
Stable mutants - when a new residue is Hydrophilic	5(17.9%)	19 (24%)
Stable mutants - when a new residue is Positive	4 (14.2%)	7 (8.8%)
Stable mutants - when a new residue is Negative	1 (3.6)	4 (5%)
**Total number of mutant proteins with higher binding affinities than WT after docking screening with drug ligands**	**18**	**0**
Mutant proteins with higher binding affinities than WT after docking screening - Hydrophobic	11	0
Mutant proteins with higher binding affinities than WT after docking screening - Hydrophilic	4	0
Mutant proteins with higher binding affinities than WT after docking screening - Positive	3	0
Mutant proteins with higher binding affinities than WT after docking screening - Negative	0	0
**Å = Angstrom**		

After docking of desired ligands against the protein binding site the data were analysed using Swissdock plugin UCFS Chimera (Supplementary Fig. S4**)** and (Supplementary Table S2). Mutant proteins showing higher binding affinities to the selected ligands than wild type (WT) were from substitutions of four residues only, namely A62, D78, S82 and K93. With the exception of A62, the remaining three residues are not hydrophobic, but referring to Table 1, these residues not only have greater distances to the atom of the PEG ligand but also they have very low contact surface to the ligand, implying that in WT these residues are not directly involved with the interaction with the ligand. This observation and the fact that they are not hydrophobic indicated that they could be easily replaced without compromising the global integrity of the protein.

Of the binding pocket residues only two (6%) are negatively charged - E14 and D78. Substitution at E14 position did not give any stable mutant, but D78 position gave 14 out of the 28 potentially stable mutants (50%). Out of these 14 stable mutants, seven were obtained when the new residues were hydrophobic **(**Supplementary Table S1**)**. From Supplementary Table S2, in the case of D78, 9 of the 14 potential stable mutants were selected as having higher affinities (after docking screening) compared to WT. Of these, 4 contained new hydrophobic residues (44%).

Only four of the binding pocket residues are positively charged. Of these H77 and K93 gave three and seven stable mutants respectively. None of the three stable H77 mutants showed higher binding affinities than WT protein, so they were not selected after docking-screening experiments with the desired ligands. Out of the seven potential K93 mutants, five are hydrophobic while two are positive **(**Supplementary Table 1**)**, except for K93L all remaining six mutants were selected after docking. K93 lies around the border of the mouth of the AgamOBP1 binding pocket (Supplementary Figs. S5 and S6) and even hydrophobic substitutions enhanced ligand binding.

S82 was predicted to have two stable mutants (S82A and S82P) with the new residues being hydrophobic **(**Supplementary Table S1**)**. They were both selected as they showed higher affinities to the tested drug ligands compared to WT after docking-screening experiments.

The main binding pocket (active site) of AgamOBP47 is made up of 60 residues, of which 39 are hydrophobic (65%), 13 are hydrophilic (21.6%), 5 are positive (8.3%), and 3 are negative (5%). Out of those 60 residues, 18 gave stable mutants (Table 2). Of these, seven are hydrophobic residues (39.9%), five are hydrophilic (27.8%), three are positively charged (16.6%), and three are negatively charged (16.6%) as shown in Table 2. From these potential residues 79 stable mutations could be identified (Table 2). However, when docking-screening analysis was done none of these gave higher affinities to the tested ligands than WT (Table 2). The docking results further suggested that the active site of AgamOBP47 was greatly conserved and although some of its residues were able to produce stable mutant proteins, these substitutions did not enhance the binding affinities and in fact all mutants showed lower binding energies towards the docked ligands (Table 2).

### Protein expression and characterisation

**Figure 1:**
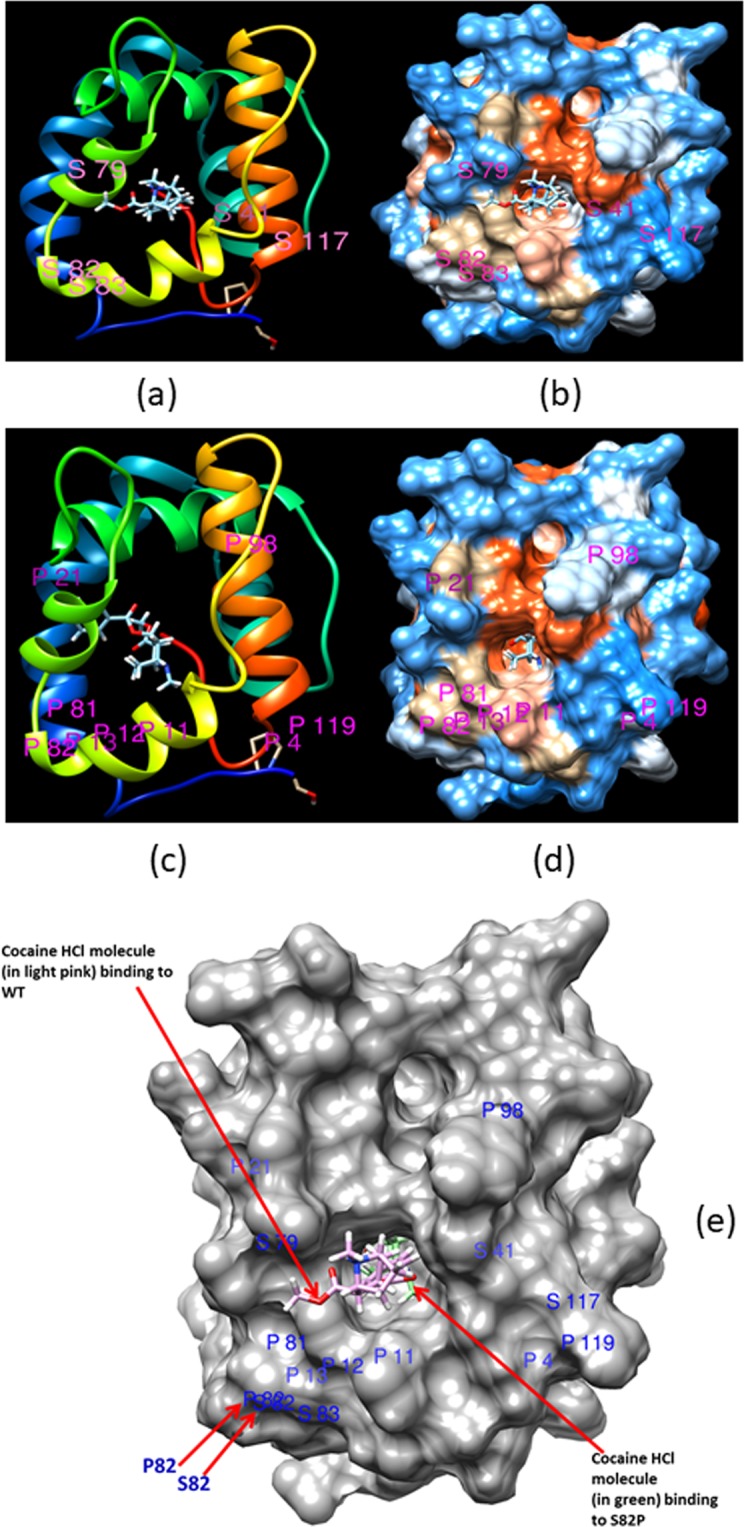
Binding of a cocaine-HCl molecule to the binding site of: (**a,b**) Wild type (WT) mosquito- AgamOBP1, and (**c,d**) AgamOBP1_mutant S82P. In (**a**) cartoon representation of the X-Ray structure and (**b**) hydrophobicity surface representation, the cocaine-HCl molecule shows does not fit into the WT AgamOBP1 binding pocket, but in (**c**), cartoon representation, (**d**) hydrophobicity surface representation, after substitution of residue number 82 Serine (S) with Proline (P) to produce the mutant S82P, the cocaine-HCl molecule can now fit into the binding pocket. (**e**) Structural superposition of (**b**) and (**d**), where cocaine-HCl molecule in (b) is shown in light pink, while cocaine-HCl in (**d**) is shown in green. Residue S82 from (**b**) and residue P82 from (**d**) are labelled.

**Figure 2:**
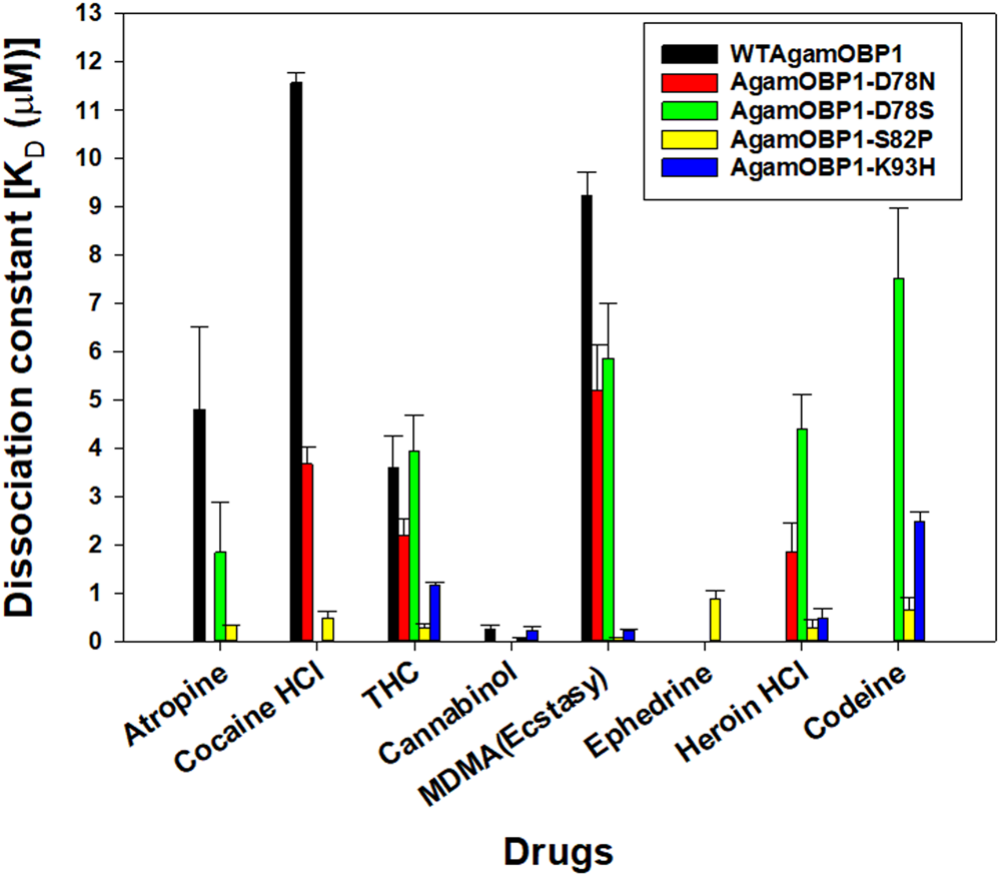
Dissociation constants of WTAgamOBP1 and its mutant variants AgamOBP1-D78N, AgamOBP1-D78S, AgamOBP1-S82P, AgamOBP1-K93H toward the tested ligands Atropine, Cocaine-HCl, THC, Cannabinol, MDMA, Ephedrine, Heroin-HCl, Codeine (data in Table 3), determined experimentally in solution. Error bars represent standard deviations from 3 replicate measurements.

From the list of mutants identified, we chose to express 4 mutants of OBP1 (D78N, D78S, K93H, S82P) and WT with a 6His N-terminal (sequences shown in Supplementary Fig. S7a). The rationale for these choices were based on better affinity than WT for the selected ligands, together with assessment of conformational stability if an amino acid was substituted. The AgamOBP1 active site is highly hydrophobic and conserved, in fact 11 of 18 potential mutants identified (Supplementary Table 2) were hydrophobic in nature. A decision was made to choose those mutants where the new amino acid was not hydrophobic as they may have better potential to induce meaningful changes in binding affinity to the ligands of interest. Position A62 gave only one mutant A62L (hydrophobic to hydrophobic) so this was discarded. At position D78 (D is negative) we had 9 possible mutants and of these 5 were when the new amino acid was not hydrophobic (D78N, D78Q, D78R, D78S, D78T). D78N was the most potent against the ligands of interest (Supplementary Table 2 last column) and was chosen for expression. While the cocaine-HCl molecule could not completely access the binding pocket of the WT protein it can do so with mutants D78N and S82P (Fig. 1) so these were selected. Another mutant we chose as a control at this position was D78S, as according to our selection criterion (Supplementary Table 2 last column) it was the least potent out of the 18 selected potential mutants and indeed this was confirmed by the experimental data (Table 3 and Fig. 2). At position S82 there are two possible mutants S82A and S82P and they are of similar potency (Supplementary Table 2 last column), although P is less hydrophobic than A (Supplementary Table 1 last column), and as previously stated, S82P is the one of the two mutants where the cocaine-HCl molecule can completely access the binding pocket. The stability energies show that S82P [ΔG (Kcal/mol) = −0.28] is nearly one order of magnitude more stable than S82A [ΔG (Kcal/mol) = −0.03] and therefore we choose S82P for expression. The final position was K93 that gave 7 out of the 18 better mutants than wild type where two (K93H and K93R) were when the new amino acid was not hydrophobic. Although K93R is potentially better than K93H (Supplementary Table 2 last column), as K93H [ΔG (Kcal/mol) = −0.3] is more stable than K93R [ΔG (Kcal/mol) = −0.1] we expressed K93H.

**Table 3:** Experimental binding constants determined in solution.

Ligand	Dissociation constants (K_D_ µM)		AgamOBP1_D78S	AgamOBP1_S82P	AgamOBP1_K93H
	WT_AgamOBP1	AgamOBP1_D78N			
**1-NPN**	**0.49 ± 0.1**	**1.9 ± 0.5**	**2.03 ± 0.13**	**0.22 ± 0.1**	**0.83 ± 0.24**
Atropine	4.81 ± 1.72	NA	1.84 ± 1.03	0.34 ± 0.002	NA
Cocaine HCl	11.57 ± 0.22	3.67 ± 0.35	NA	0.48 ± 0.14	NA
THC	3.59 ± 0.66	2.19 ± 0.36	3.94 ± 0.73	0.28 ± 0.07	1.17 ± 0.05
Cannabinol	0.26 ±0.08	ND	ND	0.06 ± 0.02	0. 22 ± 0.08
MDMA(Ecstasy)	9.24 ± 0.48	5.19 ± 0.95	5.86 ± 1.15	0.08 ± 0.01	0.23 ± 0.02
Ephedrine	NA	NA	NA	0.88 ± 0.17	NA
Heroin HCl	NA	1.86 ± 0.58	4.39 ± 0.72	0.27 ± 0.17	0.47 ± 0.21
Codeine	NA	NA	7.51 ± 1.47	0.65 ± 0.27	2.48 ± 0.19
**NA = No binding observed**.					
**ND = Was not determined**.

These proteins were expressed and purified as described by others^21,35^, purity checked by SDS gel electrophoresis (Supplementary Fig. S7b**)** and binding constants characterised using a fluorescent competitive binding assay^35^ against the ligands of interest as well as control related substances atropine, cannabinol, cannabidiol and codeine. Figure 2 and Table 3 summarise the dissociation constants (K_D_ in µM) obtained against these ligands in solution. S82P proved to have improved affinity (1/K_D_) to all ligands tested over the WT and had different affinities to all of the ligands, with the highest affinity to cannabinol.

From the experimentally determined binding constants (Table 3) AgamOBP1 mutants D78S and S82P displayed respectively 3 and 13-fold higher affinities (1/K_D_) compared to the WTAgamOBP1 towards atropine, but S82P had the highest affinity towards atropine compared to all proteins. From all five OBP variants only two proteins D78N and S82P recognised cocaine, with almost 3 and 25-fold higher affinities respectively compared to the WT. These data are consistent with the docking results that showed that while the cocaine molecule could not completely access the binding pocket of the WT protein it does with these two mutants D78D and S82P (Fig. 1). All OBPs recognise THC and the mutant S82P has the highest affinity towards THC compared to all OBPs tested (13-fold higher compared to WT). As a control cannabinol (an analog of THC) was included. Binding constants for cannabinol could be determined only for WT, S82P and K93H with S82P having the highest binding affinity that was about 4-fold higher compared to WT. S82P and K93H have respectively almost 105 and 36-fold higher affinities towards MDMA compared to WTAgamOBP1. The affinities of these two mutants towards MDMA are also higher than all other mutants tested. The data showed that only mutant S82P can recognise ephedrine with a K_D_ of 0.88 µM.

### Comparison between experimental and theoretical data

AgamOBP1 has one continuous channel running from one side of the molecule to the other. The pocket is a channel with two mouth openings (Supplementary Fig. S5). In WT (Supplementary Fig. S6a) the ligands are largely stack at the entry mouth and interact with the residues surrounding that area. While *in silico* models make assumptions about the rigidity of the protein structures and must be cautiously interpreted, from the theoretical docking experiments, in the case of the mutants like S82P (Supplementary Fig. S6a), the pocket appears more flexible and ligands like atropine and THC are able to access the binding pocket. In the case of heroin (Supplementary Fig. S6b–d), which is a larger molecule compared to the other drugs, this managed to slightly stretch the entry mouth. In both cases it is just one of the two carboxylic moieties of the heroin molecule that are able to penetrate the mouth openings of WT and mutant, but in neither of these two cases could the heroin molecule fully access the binding pocket, giving very high positive binding energies (WT was 32.3 Kcal/mol while S82P was 36.03 kcal/mol) indicating a very weak affinity of heroin to the proteins. There is no interaction between cocaine and S82 in the WT protein from the docking experiments. From the docking experiments with mutants, it was found that two residues greatly influence the entry of cocaine into the binding pocket. The distance between cocaine atom O4 and to S82 (in WT) atom CA is 12.192 Å (Supplementary Fig. 8a,b), while the distance between the same cocaine atom O4 to P82 (in S82P) atom CD was found to be 15.507 Å. The results showed that in the WT, residue S82 is very close to the cocaine molecule at the entry mouth preventing it from entering the pocket and the ligand does not interact with this residue. However, in the mutant S82P the residue is pushed away from the mouth entrance allowing the cocaine molecule to enter the pocket as confirmed by the experimental binding data. Also, for residue H77 in the WT the distance between the cocaine O4 atom and the CA atom of H77 is only 6.176 Å, preventing cocaine from entering the pocket. On the other hand, in the mutant S82P, the distance between the cocaine O4 atom and the H77 CA atom is 10.235 Å, so it is pushed away from the mouth entrance allowing cocaine to enter the pocket.

In S82P **(**Supplementary Table S3**)** the active site mouth molecular surface area is larger by 0.43 Å^2^ compared to that of the WT protein, at the same time in S82P **(**Supplementary Table S3**)** the mouth molecular surface length is longer by 0.62 Å compared to that of WT protein. These data can explain why the cocaine molecule could not fit and penetrate the binding pocket in the WT protein (Fig. 2), but it was able to fit and penetrate the binding pocket of the mutant protein S82P. These data agree with the description given above and highlighted in **(**Supplementary Fig. S8**)**.

Experimentally some binding affinity was observed in WT for heroin (in contrast to that predicted by the modelling tools) but higher affinities were found for the mutants particularly S82P. A possible explanation could be that heroin bound to the mutant proteins via a slightly different route to the traditional one in WT. This is not surprising particularly for S82P, a mutant that showed higher affinities toward all drugs tested experimentally when compared to the other proteins (as described above). It also has to be noted that the docking tool used predicts multiple binding modes (clusters) and ranks them according to their energy values, the ranking preferred those binding modes within the traditional and main binding pockets and ranked them by giving them low energies values corresponding to higher affinities.

The mutant D78S generally displayed lower affinities experimentally towards drugs compared to WTAgamOBP1, this is consistent with the docking energies values presented in Supplementary Table S2, where the binding of target ligands to this protein were less energetic compared to the other three mutants tested experimentally.

### Biosensor development

**Figure 3:**
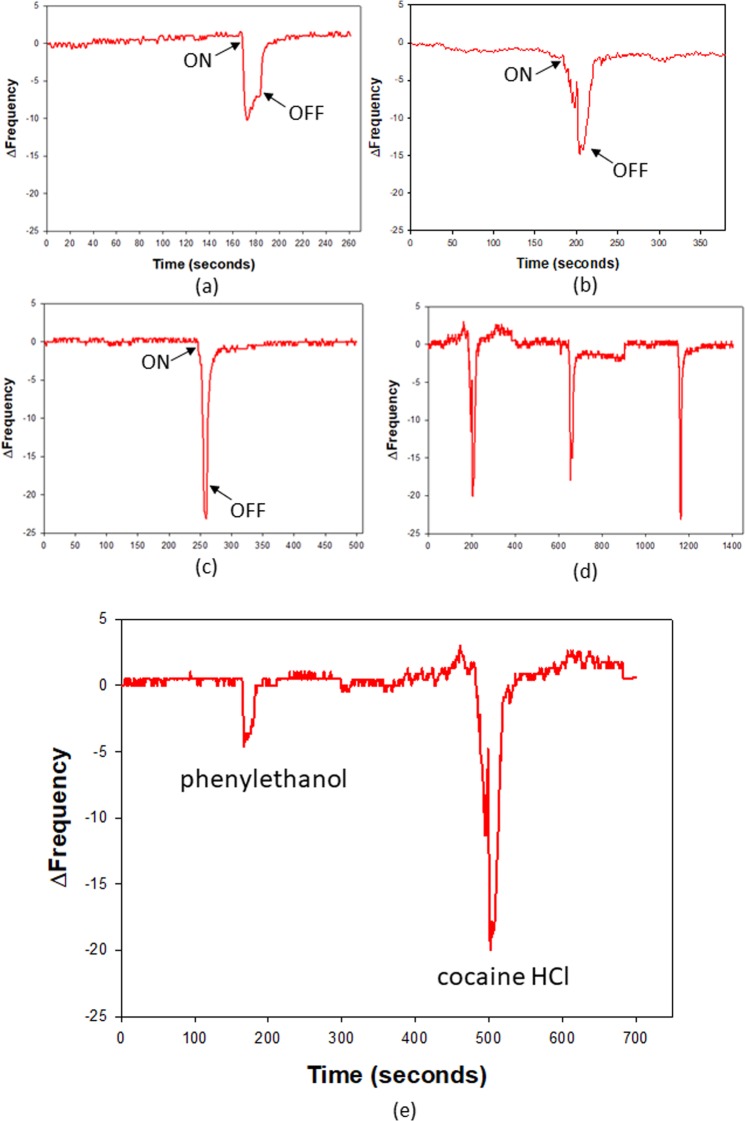
Responses to saturated cocaine vapour by (**a**) WTAgamOBP1, (**b**) AgamOBP1_K93H, (**c**) AgmOBP1_S82P, (**d**) Repeated responses to saturated cocaine-HCl vapour by AgamOBP1_S82P; Measurements were repeated twenty times, as an example three repetitions are shown in this figure. In (**a**), (**b**), and (**c**) ‘ON’ = Start of measurement while ‘OFF’ = End of measurement. (**e**) Test of selectivity showing responses to saturated 2-phenylethanol vapour (left) (SVP 1.31 × 10^−4^ atm) and saturated cocaine vapour (right) (SVP 1.84 × 10^−11^ atm) by AgamOBP1_S82P.

**Figure 4:**
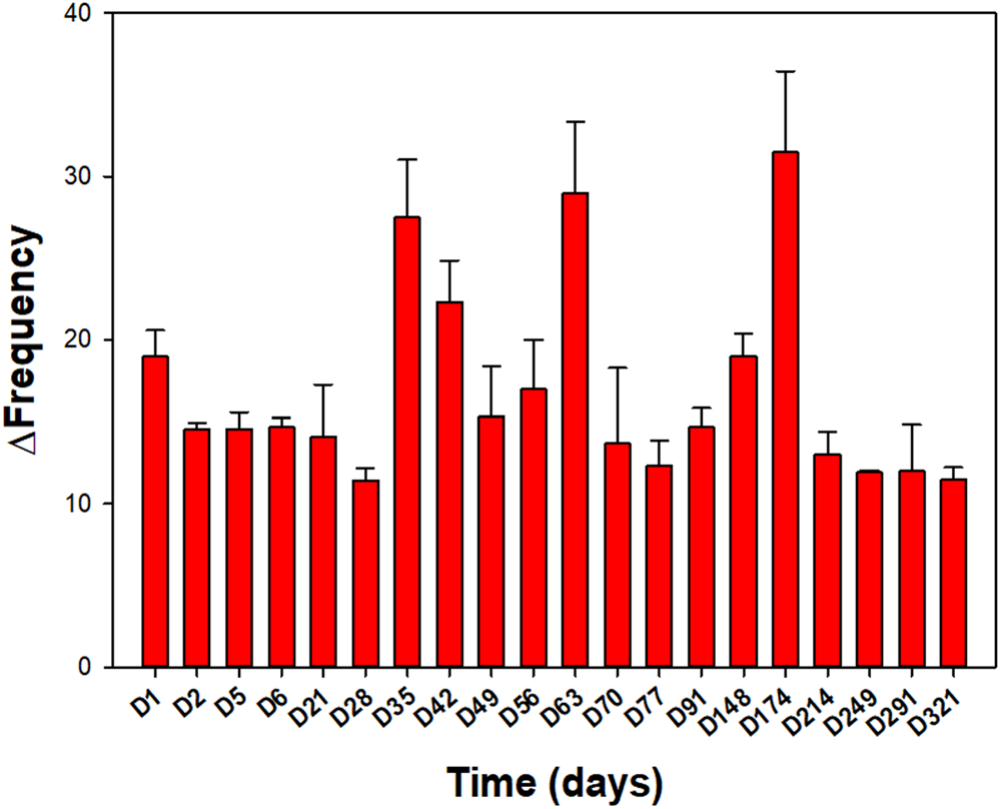
Reponses recorded from the AgamOBP1_S82P sensor to repeated presentations of saturated cocaine-HCl vapour over a period of 10 months at room temperature. Variations are largely due to differences in saturated vapour concentrations due to variation in temperature over this period. The data indicate that the proteins continue to respond to the analyte over long periods without signs of degradation.

The next stage was to test whether a biosensor could be constructed that could detect the analytes in the vapour phase. Proteins were immobilised on to 20 MHz Quartz Crystal Microbalances (QCMs) using self-assembled monolayer techniques^36^ and the resulting biosensors were exposed to pulses of saturated analyte vapour (confirmed by headspace GC-MS (Supplementary Fig. 9)). Because of the low vapour pressures of the substances tested (Supplementary Table 4), no attempt was made to dilute these further. While the majority of the analytes were undetectable, it was interesting to find that the sensors were able to sensitively respond to saturated analytical grade cocaine hydrochloride vapour (SVP 1.84211 × 10^−11^ atm at 20 °C)^37^ (Fig. 3). The WT protein consistently had a lower response to the saturated vapour (Fig. 3a) than mutants K93H (Fig. 3b), and S82P (Fig. 3c,d). 2-Phenylethanol has poor binding affinity to the protein in solution and was tested as a control. The S82P biosensor was shown to have much greater selectivity to cocaine HCl vapour than to 2-phenylethanol vapour as shown in Fig. 3e. The vapour pressure of 2-phenylethanol is 1.31 × 10^−4^ atm at 20 °C. It took almost five orders of magnitude more of 2-phenylethanol to give just a quarter of the cocaine response (Fig. 3e). The stability of these OBP biosensors while immobilised on QCMs was monitored over ten months under laboratory operating conditions. Despite small daily variations in response due to variations in the concentrations of saturated vapour generated at ambient temperatures these sensors were still able to continue to detect the target analyte vapour over this period (Fig. 4).

## Discussion

Agam-OBP1 is associated with perception of the physiologically relevant compound indole with an *in vitro* measured K_D_ of 2.3 µM^22^ and while this may be a natural ligand for this protein, binding experiments by other researchers using fluorescence displacement assays indicate that the affinity is low for indole compared to other ligands^21^. This has prompted interesting discussions about the possible formation of dimers in solution where ligand-binding experiments will be dependent on the degree of asymmetry of the dimer and the dissociation constant for the equilibrium monomer/dimer. Here we have focussed on the monomeric form of the protein, with the observation that the nature of the binding pocket also allows it to bind a range of ligands of different sizes. We show that single amino acid substitutions in the binding pocket of this OBP (modelled *in silico* and then tested experimentally on expressed proteins) cause dramatic changes in the binding affinity to target ligands that were structurally completely different from indole. On the other hand, OBP47 proved not to be useful in this case for the targeted ligands. *In silico* modelling proved to be useful to document the binding pocket residues that had the closest interactions with the target ligands. As observed by Chiappori^28^, the mutagenesis method that was adopted does not require previous knowledge of functional or structural role of involved residues so can therefore be applied to explore new binding features. Here, docking of the selected ligands to the mutants created *in silico* allowed some preselection of suitable proteins to be expressed and tested experimentally. Assumptions made by the modelling programs means that results obtained should be critically examined. Challenges faced in this case were choice of an energy threshold that could be used to determine whether binding of a ligand to a mutated protein could be judged as better or worse than wild type, and an arbitrary threshold was chosen. Experimentally, the affinity constants measured deviated from those that would be predicted from the binding energies calculated, but this is a limitation based on the assumptions used by the modelling tools, as well as the fluorescent displacement assay that was used to determine experimental dissociation constants. Apart from heroin, the ranking of the measured binding constants agreed with that predicted by the models. The OBP1 mutants showed a range of experimental affinities to the target ligands, each mutant having a different pattern of binding activity, indicating a broad spectrum of selectivity. This can be usefully utilised by combining these proteins into an array of biosensors - in this case the pattern of binding affinities measured would be sufficient to discriminate between one drug and another in analogy to how “electronic nose” data are processed^38^. For most of the substances of interest, these would need to be analysed in solution as the vapour pressures are extremely low. The expected sensitivity of such an array would be in the range of 10^−6^–10^−8^ M based on the dissociation constants measured. The combinatorial approach taken allows flexible design of new biosensors based on inherently stable protein scaffolds taking advantage of the stable tertiary structure of odorant binding proteins.

Detection of vapours by immobilised OBPs in the absence of an aqueous environment is also clearly illustrated here and by others^14,39,40^. The detection of cocaine hydrochloride vapour at extremely low concentrations using a simple quartz crystal microbalance transducer indicates that there is scope for creating arrays of OBPs that are tailored to detect compounds of interest that are volatile, with potential for security applications or for forensic drug analysis. The stability of immobilised OBPs in air (Fig. 4) is of great interest for future gas sensing applications as they are able to function as binding proteins without being immersed in an aqueous environment as is normal for other types of biosensors.

## Materials and Methods

### Reagents

All reagents used were of analytical grade purchased from Sigma-Aldrich, Fisher Scientific, or VWR. Genes were custom synthesised at Eurofins MWG GmbH, Ebensburg, Germany. The JLMQ USB interface for 4 QMB sensors (JLM Innovation GmbH, Tuebingen, Germany) was used as a readout system for 20 MHz quartz crystal microbalances with gold coated electrodes fabricated by IMM-CNR, Italy.

### *In silico* mutagenesis

The X-ray structures of mosquito *Anopheles gambiae* OBP (AgamOBP1)^31^ was used as a template for *in silico* mutagenesis experiments. The LPC/CSU (Weizmann, AC) server^41^ was used to identify binding pocket residues that interact with the targeted ligands. *In silico* single amino acids replacement was initially carried out using the KINARI MUTAGEN server^33^ to identify residues that are feasible to mutate, this was followed by single amino acid replacement to identify stable mutations around the ligand binding pocket using a program called PoPMuSiC (Prediction of Proteins Mutants Structural Changes)^34,42^. The identified stable mutants were then constructed and visualised using molecular graphics software PyMOL (Schrödinger). For Anopheles gambiae OBP47 (AgamOBP47) its X-ray structure (PDB ID: 3PM2)^32^ was used as a template for *in silico* mutagenesis experiments. Unlike that of AgamOPBP1, the X-ray structure of AgamOBP47 does not have any ligand bound to the protein, therefore the Computed Atlas of Surface Topography of proteins (CASTp) web server^43^ was used to identify the potential binding pockets in this protein.

### The procedures for using the LPC/CSU Weizmann server, CASTp server, KINARI-Mutagen  software and PopMuSiC server

These tools can be easily used by following simple instructions provided within the servers. The LPC/CSU server has been designed to assist the molecular biologist in automated analysing of interatomic Ligand-Protein Contacts (LPC) and Contacts of Structural Units (CSU) in proteins and visualising them. For this work the LPC option was selected and the protein X-ray structure was uploaded from PBD. Analysis was started and the server retrieved all the ligands associated with the PBD structure and the ligand of interest was selected for LPC analysis^31^. The results page from LPC analysis gives seven different tables each with a different type of analysis, for this work the important table was the one listing the residues in contact with the ligand^41^.

### Computed atlas of surface topography of proteins (CASTp)

Computed Atlas of Surface Topography of proteins (CASTp) provides an online resource for locating, delineating and measuring concave surface regions on the three-dimensional structures of proteins. These include pockets located on protein surfaces and voids buried in the interior of proteins. The measurement includes the area and volume of pocket or void calculated analytically using a solvent accessible surface model (Richards’ surface) and a molecular surface model (Connolly surface). CASTp includes a graphical user interface, flexible interactive visualization, as well as on-the-fly calculations for user uploaded structures accessed at http://cast.engr.uic.edu.

### KINARI software for kinematic and rigidity analysis of proteins

KINARI is a suite of tools for calculating and analyzing the rigidity and flexibility of biomolecules and the KINARI-Mutagen tool was used for this work^33^. KINARI-Mutagen performs *in silico* mutation experiments on protein structures from the Protein Data Bank and analyzes their rigidity. A Jmol-scripted embedded visualizer displays the rigidity results for each mutant and several plots are generated to help the user determine which mutation affected most the protein’s rigidity. Residues whose mutation affects the rigidity of the protein can be inferred to be critical. To generate a mutation at a user-specified residue, KINARI-Mutagen removes hydrogen bonds and hydrophobic interactions from the protein’s molecular model (Curation). This is called an excision and is structurally equivalent to substituting a residue with a glycine. The X-ray structures of *Anopheles gambiae* OBP (AgamOBP1) were curated and the binding pocket residues that interact with ligands that were identified by LPC analysis above were mutated, followed by rigidity analysis. One of the plots generated is “The Largest Rigid Cluster” and SASA (Solvent Accessible Surface Area), vs. Residue plot together with a table of data used by the server to generate that plot. The table provides information about the amino acids that were *in silico* mutated, along with the rigidity information for each mutant. AgamOBP1 binding pocket residues where rigidity was not affected were selected as potential residues for generation of mutant variants.

### Prediction of protein mutants stability changes (PoPMuSiC)

The PoPMuSiC software is a tool for the computer-aided design of mutant proteins with controlled stability properties^34,42^. It evaluates the changes in stability of a given protein or peptide under single-site mutations, based on the protein’s structure. Three modes are available; Systematic, Manual or File. The Systematic tool evaluates the stability changes resulting from all possible mutations and returns a report containing a list of the most stabilizing or destabilizing mutations, or of the mutations that do not affect stability. The Manual tool predicts the stability change resulting from one or more given mutations. The File tool predicts the stability changes resulting from a list of mutations specified by the user in an uploaded file. For this work the systematic tool was used to evaluate the stability changes resulting from all possible mutations of AgamOBP1 (where each residue is mutated with each of the 20 amino acids minus its own). From the resulting file we concentrated on the mutations of the binding pocket residues identified by LPC and KINARI-Mutagen tools above. All mutations that were identified as stabilising were selected and recorded and used in the docking screening process below.

### Docking screening experiments

The Swissdock server provided by the Swiss Institute of Bioinformatics (http://swissdock.vital-it.ch/docking) using “EADock DSS software” was used for the docking of the ligands into the generated mutants and WT. The resulting docking predictions were viewed and analysed using the Swissdock server plugin in UCSF Chimera. Selected mutants were designed as 6-His tag constructs and the gene synthesis was ordered from Eurofins MWG operon.

### Bacterial expression of insect OBPs

For the expression of recombinant insect OBPs, *E. Coli* BL21 (DE3) pLysS competent cells were transformed with a plasmid vector (pET-9d for AgamOBP variants) containing appropriate OBP sequences. The culture was grown in a shaking incubator set at 300 rpm at 37 °C and when the culture reached OD (600 nm) of 0.8 it was induced with 0.4 mM isopropyl β-D-1-thiogalactopyranoside (IPTG). Cells were grown for further 3 hours in the same conditions, harvested by centrifugation at 4000 rpm for 30 minutes at 4 °C and the cell pellet was stored overnight at −80 °C. The cell pellet from 1 L culture was suspended with 10 ml of extraction buffer: 50 mM Tris-HCl, pH 7.4 + 0.5 M NaCl + 1 mM phenylmethanesulfonyl fluoride (PMSF) protease inhibitor and then sonicated 4 × 3 minutes (with 3 minutes breaks in between) at a 40% duty cycle. After centrifugation at 15000 rpm for 30 minutes, the pellet was washed with 10 ml of the same buffer. The OBP pellet consisted of inclusion bodies that were solubilised with 10 ml of 8 M urea in 50 mM Tris-HCl, pH 8 and incubated under agitation at room temperature for 30 minutes. At this point 1 mm dithiothreitol (DTT) was added and the sample was further incubated with agitation at room temperature for 60 minutes. The sample was diluted to 100 ml (ten-fold) with 50 mM Tris-HCl, pH 7.4 buffer, and then dialysed against the same buffer for 24 hours at 4 °C changing the dialysis buffer three times. After dialysis the sample was centrifuged at 4000 rpm for ten minutes to remove any aggregates. The 6His-tagged OBPs were then purified using HisPrep FF 16/10 (GE Healthcare). The equilibration buffer was 50 mM Tris-HCl, pH 7.4 + 500 mM NaCl + 35 mM imidazole and elution buffer 50 mM Tris-HCl + 500 mM NaCl + 500 mM imidazole. To each 6His-tagged insect OBP sample 35 mM of imidazole was added prior to column loading in order to attain highest purity. Protein-containing fractions were analysed on SDS-PAGE; following this, if protein was not clean enough, it was purified further by gel filtration on Sephacryl-100 or Superose-12 with 50 mM Tris-HCl, pH 7.4. The OBP proteins were finally stored in 50 mM Tris-HCl, pH 7.4. The procedures were carried out according to standard protocols previously adopted for insect OBPs^31^.

### Delipidation of the purified insect OBPs

Sephadex LH-20 resin powder was hydrated with 50 mM acetate buffer pH 4.5 by tumbling at room temperature for 3 hours and the slurry was spin washed twice with the same buffer. The slurry was re-suspended in the same buffer at 25% buffer and 75% slurry (volume/weight). The pH of the protein samples was reduced to 4.5 with acetic acid. Then 1 part (slurry) was mixed with 5 parts protein sample and this was stirred or tumbled at 4 °C for 90 minutes. The sample was centrifuged and the supernatant containing protein was filtered (with a 0.8 µm filter). Finally, this was dialysed with 50 mM Tris-HCl, pH 7.4.

### Fluorescence measurements

Emission fluorescence spectra were recorded using a Perkin Elmer LS55 or LS50 Luminescence spectrometer instrument at 25 °C in a right-angle configuration, with a 1-cm light path quartz cuvette and 5-nm slits for both excitation and emission.

### Fluorescence binding assays

The dissociation constants (K_D_) of the OBPs against target drugs were determined in competitive binding measurements. Firstly, the K_D_ of the fluorescence probe N-phenylnaphthalene-1-amine (1-NPN) against the protein was determined. To a 1 µM solution of the protein in 50 mM Tris-HCl, pH 7.4, aliquots of 1 mM 1-NPN in methanol were added to achieve final concentrations of 0.2–12 µM. The probe was excited at 295 nm and emission spectra were recorded between 337nm-450nm (OBP protein–NPN complex peak 405–410 nm). The interaction between the protein and the probe was monitored by recording the fluorescence intensity increase upon addition of 1-NPN aliquots. The experiments were replicated at least three times. The dissociation constant (K_D_) of the OBP-protein-NPN complex was calculated from the binding curve by non-linear-least-squares fit of the experimental data using the equation y = B_max_ [NPN]/(K_D_ + [NPN]) where [NPN] is the concentration of the free probe, y is the specific binding derived by measuring fluorescence intensity and B_max_ is the maximum amount of complex formed at saturation. The computer program used to fit the data was SigmaPlot 12.3 (Systat software, Inc. USA). Once the K_D_ of 1-NPN was determined, the K_D_ of the target analytes were measured in competitive binding assays, recording the fluorescence intensity decrease upon addition of aliquots of 1 mM analytes to give final concentrations between 0.5–16 µM to a solution containing 1 µM protein, and 5 µM 1-NPN in 50 mM Tris-HCl, pH 7 4. K_D_ of the competitor analytes were calculated from the corresponding IC_50_ values (concentrations of the competitor analytes giving half of the initial fluorescence intensity value of 1-NPN) using the equation: K_D_ = [IC_50_]/(1 + [NPN]/K_NPN_), [NPN] being the free concentration of 1-NPN and K_NPN_ being the dissociation constant of the protein-probe complex determined^35,44^.

### OBPs biosensor development

OBPs were immobilized on Quartz Crystal Microbalances (QCMs) using a self-assembled monolayer^39^. The gold surface of QCMs was cleaned by dipping the crystal into Piranha solution (1:3: 30% H_2_O_2_: H_2_SO_4_) for few minutes to remove any organic residues from the surface. The QCMs were then rinsed with ddH_2_O and ethanol and dried with a nitrogen air stream. Then the QCMS were dipped into a solution of 10 mM thioctic acid (TA) in ethanol for 20 hours under nitrogen flow. The electrodes were then rinsed with absolute ethanol to remove unbound molecules of TA and were left to dry at air.

Activation of carboxylic acid groups of the self-assembled monolayer (SAM) was carried out using 20 µL of a solution of 180 mM ethyl(dimethylaminopropyl) carbodiimide (EDC) and 180 mM of N-hydroxysuccinimide (NHS) in 10 mM sodium phosphate buffer (pH 7), the reaction was left to continue for 2 hours at room temperature. The solution was then rinsed with ddH_2_O and dried at air. The immobilisation of proteins on the activated SAM was performed by pipetting 10 µL of OBP solution onto the gold surface, which was left for an hour at room temperature before gently rinsing with ddH2O and then dried in air. The SAM activation process and protein immobilisation process was performed on both sides of the QCMs. The concentration of the protein solution was variable for each OBP, depending on the expression and purification yields, however the concentration of the proteins for immobilisation was normally in the range of 0.5–1 mg/ml.

### Testing of the resulting biosensors with saturated vapour of the target analytes

The resulting sensors were exposed to pulses of vapours from target analytes. QCM measurements were at 22%RH at 23 °C and the baseline established with clean air flowing at 0.1 L/minute. Saturated analyte vapour was sampled for 10 seconds. To establish the stability of these OBP biosensors under laboratory operating conditions repeated measurements were carried out over a ten month period.

### GC-MS analysis of Cocaine-HCl sample

The headspace of cocaine-HCl was analysed using high precision, high sensitivity (at femtogram range) Agilent Technology 7890B GC- MSD (Triple Quadrupole) system. Headspace analysis was carried out using a PAL Sampler, incubation temperature 35 °C for 6 minutes, agitator speed 250 rpm, agitator on-time 5 s, agitator off-time 2 s, syringe volume 2.5 ml, syringe temperature 85 °C. GC Analysis - equilibration time 0.5 min, maximum temperature 250 °C, initial temperature 45 °C, hold time 0.5 min, post run 85 °C, temperature gradient 7 °C/min to 200 °C. Data acquisition used MassHunter GC/MS Software while data analysis was performed using MSD ChemStation data analysis tool. Column Agilent HP5MS, 30 m × 250 μm × 0.25 μm, carrier gas helium 1.2 ml/min.

## Supplementary information

Supplementary Material.
