# Supplementary material for: Modification of an Anopheles gambiae odorant binding protein to create an array of chemical sensors for detection of drugs
Source: Sci Rep. 2020 Mar 3;10:3890. doi: 10.1038/s41598-020-60824-7 (PMC7054253; doi:10.1038/s41598-020-60824-7)
Supplement: Supplementary file 1 — Supplementary Material. [file 41598_2020_60824_MOESM1_ESM.docx]

**Modification of an *Anopheles gambiae* odorant binding protein to create an array of chemical sensors for detection of drugs**

**Khasim Cali, Krishna C. Persaud^*^**

**Department of Chemical Engineering and Analytical Science, The University of Manchester, Manchester M13 9PL, UK**

***Correspondence to** [**krishna.persaud@manchester.ac.uk**](mailto:krishna.persaud@manchester.ac.uk)

**Supplementary Information**

**Supplementary Material**


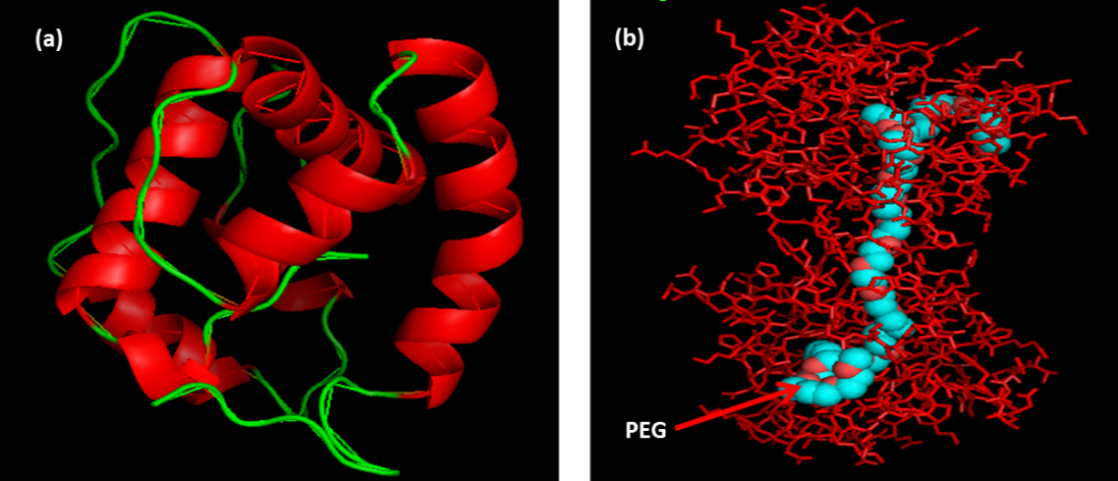


**Suppl. Material Figure S1:** The X-ray structure of AgamOBP1 (PDB ID: 2ERB). (a) is a cartoon representation Helix and Loop; (b) is a line representation of the molecule complexed with polyethylene glycol (PEG).


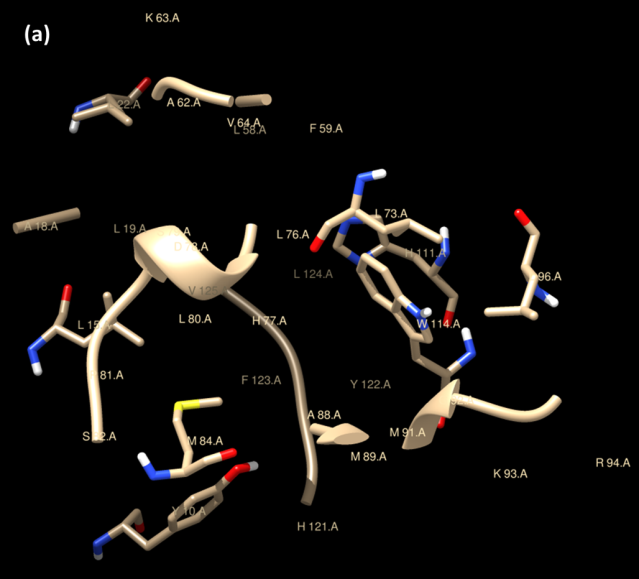

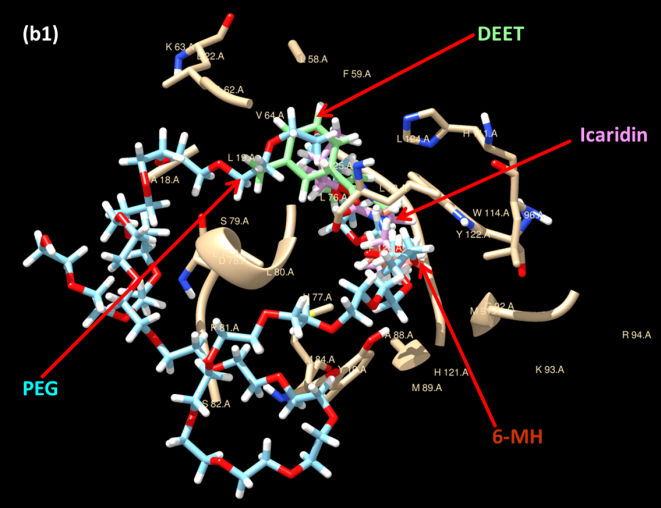


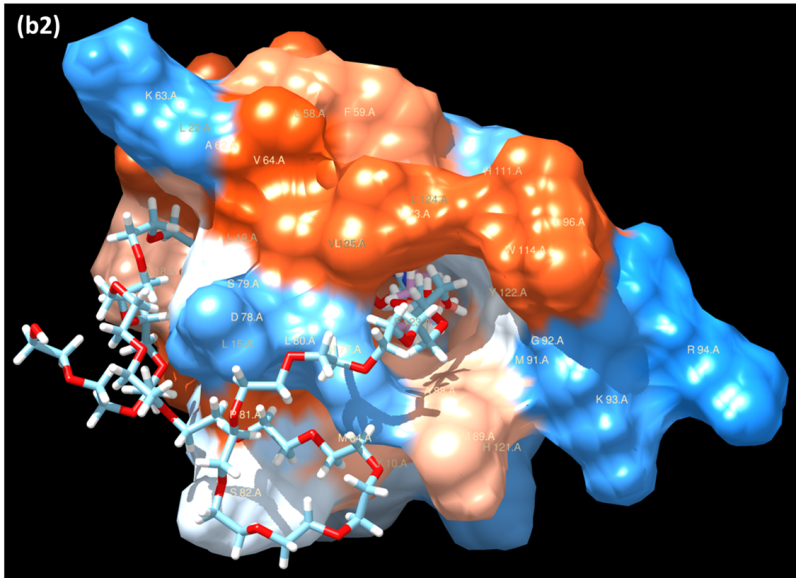


**Suppl. Material Figure S2: AgamOBP1 active site residues that interact with polyethylene glycol (PEG), N, N-diethyl-3-methylbenzamide (DEET), Icaridin, and 6-methyl-5-heptene-2-one (6-MH).**

**(a)** the active site of AgamOBP1, where all residues that interact with PEG, DEET, Icaridin, and 6-MH) are labelled**.** The interacting residues in AgamOBP1_PEG complex structure (PDB ID: 2ERB) are Y10, G14, L15,A18, L19, L22,L58, F59, A62, V64, L73,L76, H77, D78, S79, L80, P81, S82, M84,A88, M89, M91, G92,K93, L96, H111, W114, H121, Y122, F123, L124, and V125. In AgamOBP1_DEET complex structure (PDB ID: 3N7H) the interacting residues are L15, A18, L19, L22,A62,K63,L73,L76, H77, L80,A88,M89,M91,G92, K93, R94, L96, H111,W114, and. F123. In AgamOBP1_6-MH (PDB ID: 4FQT) the interacting residues are L73, L76, H77, L80, A88, M89, M91, G92, K93, L96, W114. In the case of AgamOBP1_Icaridin complex, (PDB ID: 5EL2) there is no agreement (with the exception of L76 and L80) between residues identified which are L19, L58, F59, A62, L76, S79, L80, H111,F123, L124, and V125^1^; and those found by LPC/CSU server analysis which are L73, L76, H77, L80, A88, M89, M91, G92, K93, R95, L96, W114. **(b1 and b2)** binding of PEG, DEET, Icaridin, and 6-MH, where **b1** is the cartoon representation of the X-Ray structure while **b2** is the interactive (hydrophobicity surface) representation.

1. Drakou, C. E. *et al.* The crystal structure of the AgamOBP1 Icaridin complex reveals alternative binding modes and stereo-selective repellent recognition. *Cell. Mol. Life Sci.* **74**, 319–338 (2017).


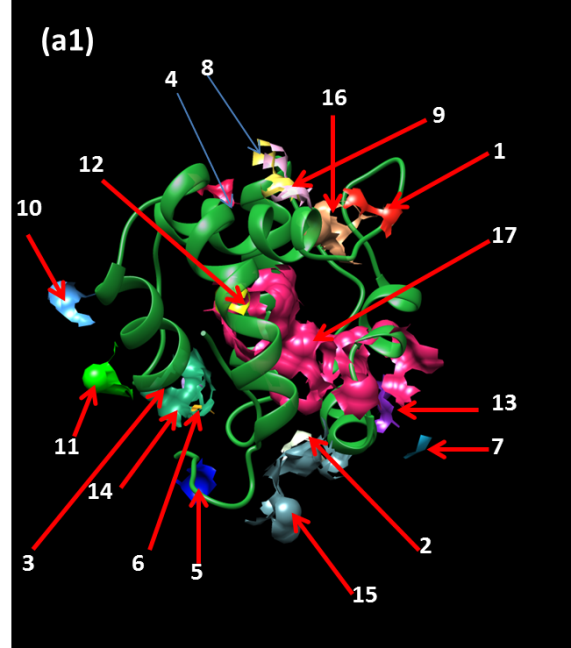

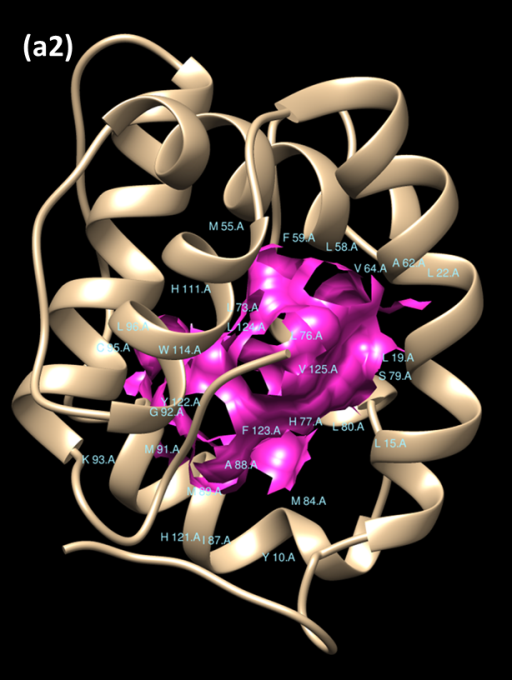


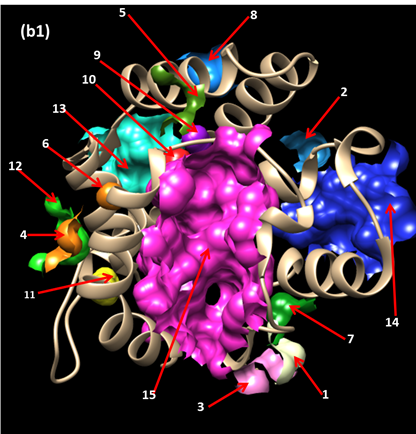

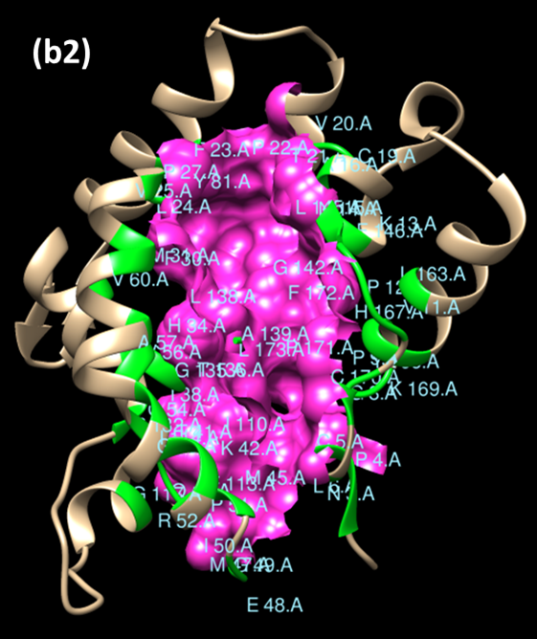


**Suppl. Material Figure S3:** Potential binding pockets in **(a1)** AgamOBP1 and in **(b1)** AgamOBP47. The X-ray structures of AgamOBP1 (PDB1D:2ERB) and AgamOBP47 (PDB ID: 3PM2) were analysed using CASTp server to identified the pockets. In **(a1)** AgamOBP1, seventeen potential pockets were identified and the main pocket (the active site) is the pocket number 17 labelled in pink. In (**b1)** AgamOBP47, fifteen potential pockets were identified and the main binding pocket (the active site) is pocket number 15 labelled in pink. In (a2 and b2) the main binding pockets (active sites) are highlighted in pink (a2) AgamOBP1 Pocket 17, and (b1) AgamOBP47 Pocket 15 and their constituent amino acid residues. The figures were rendered using UCSF Chimera software from the potential binding pockets data generated by CASTp. The characteristic features of the main binding pockets (the active sites) for both proteins are summarised in **Table 2.**


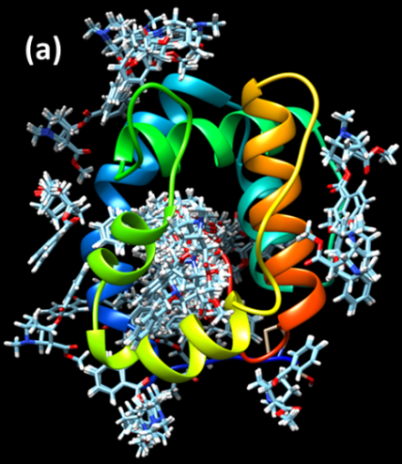

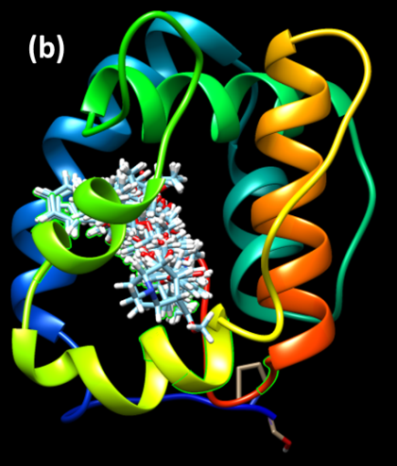

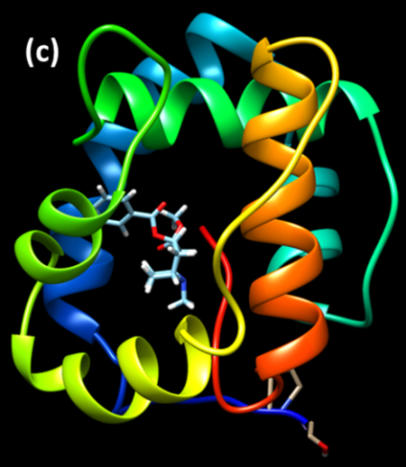


**Suppl. Material Figure S4: Visualisation of the docking outcomes using UCF Chimera:** The docking outcome for AgamOBP1_ S82P mutant with a cocaine molecule is presented as an example. (a) all predicted clusters; (b) binding pocket clusters, (c) the most energetic cluster rank (binding mode) at the binding pocket. For docking experiments, target drug molecules in addition to the fluorescence probe 1-NPN were investigated. The latter is used in displacement binding experiments to determine the affinities of binding of target ligands. The docking tool was the Swissdock server (Swiss Institute of Bioinformatics (http://swissdock.vital-it.ch/docking) which operates using “EADock DSS software”. Each of the 28 potential mutants for AgamOBP1 plus the WT was docked with each of the seven drug target ligands. During the docking procedure, the software generates many binding modes simultaneously and their energies are estimated. The binding modes with the most favourable energies were evaluated and clustered. For each docking outcome up to 42 clusters were generated, each with several rankings, a cluster is a binding mode and within it there are several ranks, each rank has slightly different energy value compared to another rank, some of these ranks are just repetitions, a much bigger energy difference occurs between one binding mode to the other. The data were visualised using the plug in “UCF Chimera”. Initially all non-binding pocket clusters were eliminated. Then the most energetic binding pocket cluster was selected and refined by eliminating any repeated ranks and (for each ligand against each protein variant) this value was then recorded in Supplementary Material Table S2.


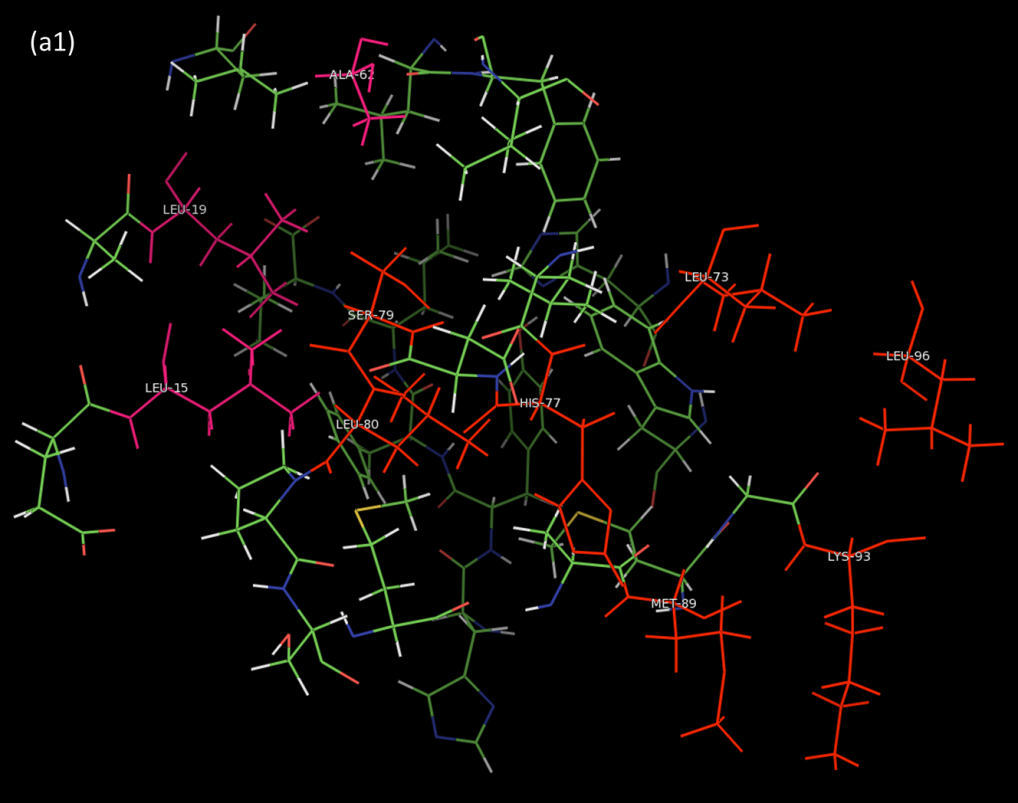


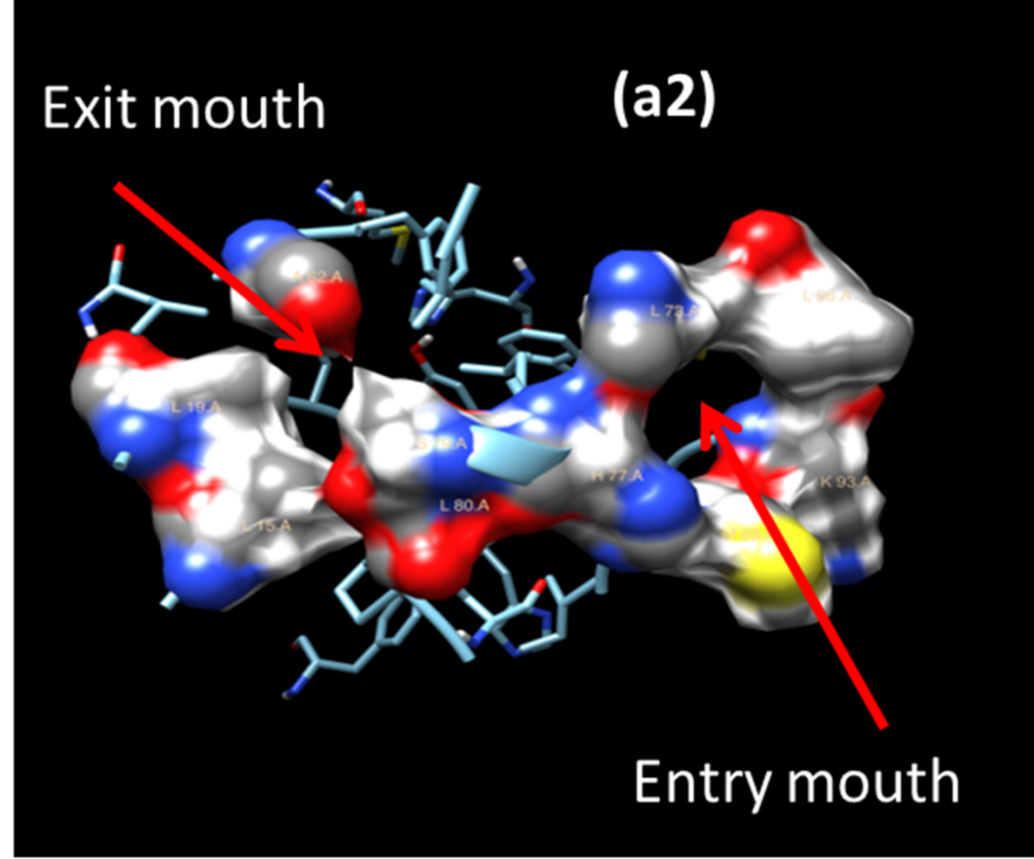


**Suppl. Material Figure S5: Mouth openings for the active site of AgamOBP1. (a1)** Mouth openings for the active site of AgamOBP1, residues making up the entry mouth are shown in red, while residues making up the exit mouth are shown in magenta, while in **(a2)** the mouths are shown interactively (hydrophobicity surface). In AgamOBP1 the active site channel has a distinct entry mouth and an exit mouth**.**  From this figure and Table 1, the residues making up the entry mouth are L73, H77, S79, L80, M89, K93, and L96, while those making up the exit mouth are L15, L19, and A62.


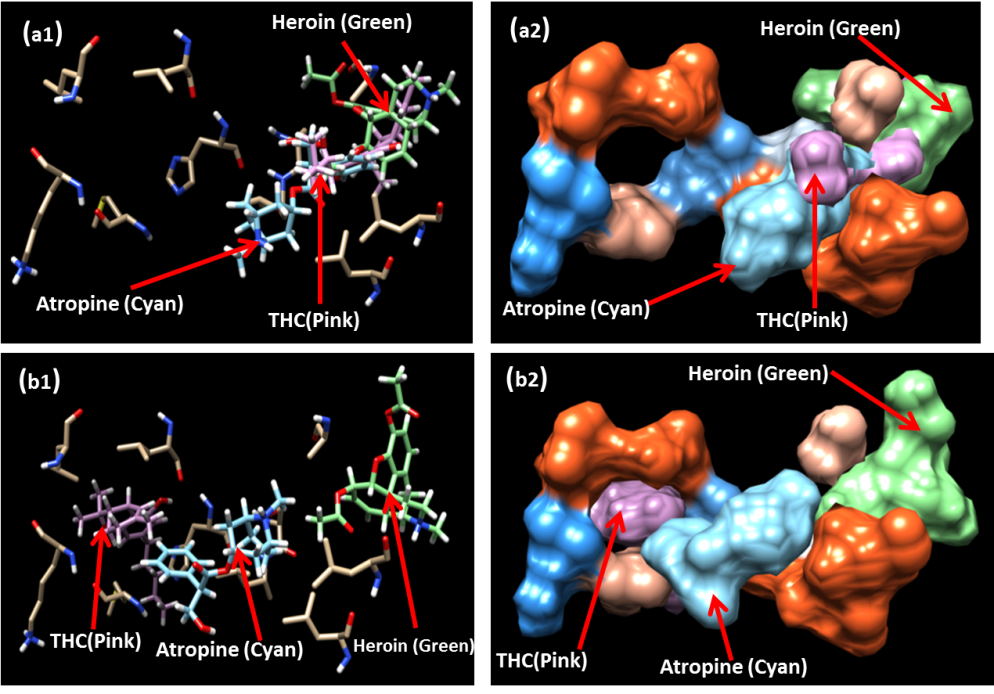


**
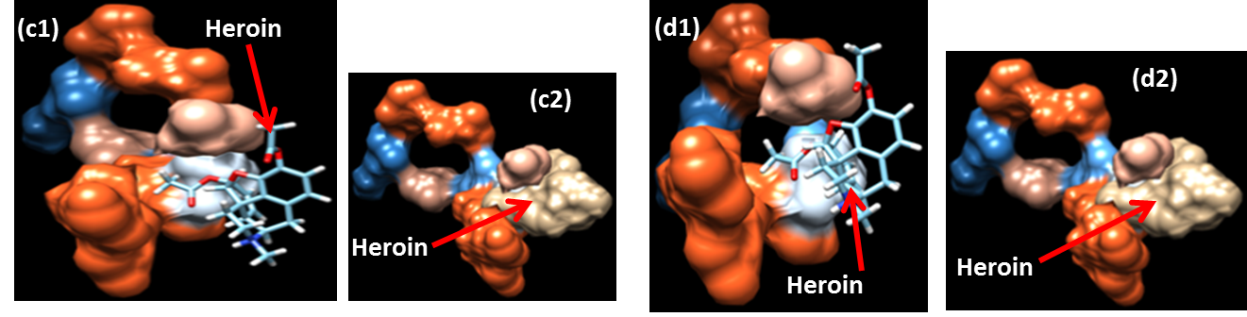
**

**Suppl. Material Figure S6: AgamOBP1 Conformational changes due to mutations: Effect on Ligand binding**. In **(a and b)** the residues at the mouth of the binding pocket are shown. For WT_AgamOBP1 **(a)**, the ligands are largely blocked at the inlet mouth and interact with the residues surrounding that area. In the case of the mutants like AgamOBP1_ S82P **(b)** the pocket now appears flexible and ligands like atropine and THC are able to penetrate fully into the binding pocket. In the case of heroin, which is a larger molecule compared to other ligands, it still follows the same route as the other ligands for WT but for the mutant the heroin molecule manages to slightly stretch the inlet mouth. In both cases it is just one of the two carboxylic moieties of heroin molecule that is able to penetrate the mouth openings of WT and mutant. The heroin molecule is unable to penetrate the binding pocket fully. In **a1** and **b1**, stick models are illustrated, while in **a2** and **b2** the mouths and ligands are shown interactively (hydrophobicity surface). In (**c** and **d**) the binding of a heroin molecule to the pocket of **(c)** WT and **(d)** AgamOBP1_S82P is shown in more detail, illustrating in **c1, c2** – failure to enter the WT binding pocket, **d1, d2** – closer proximity of a carboxylic moiety of the heroin molecule to the mouth of the S82P mutant.


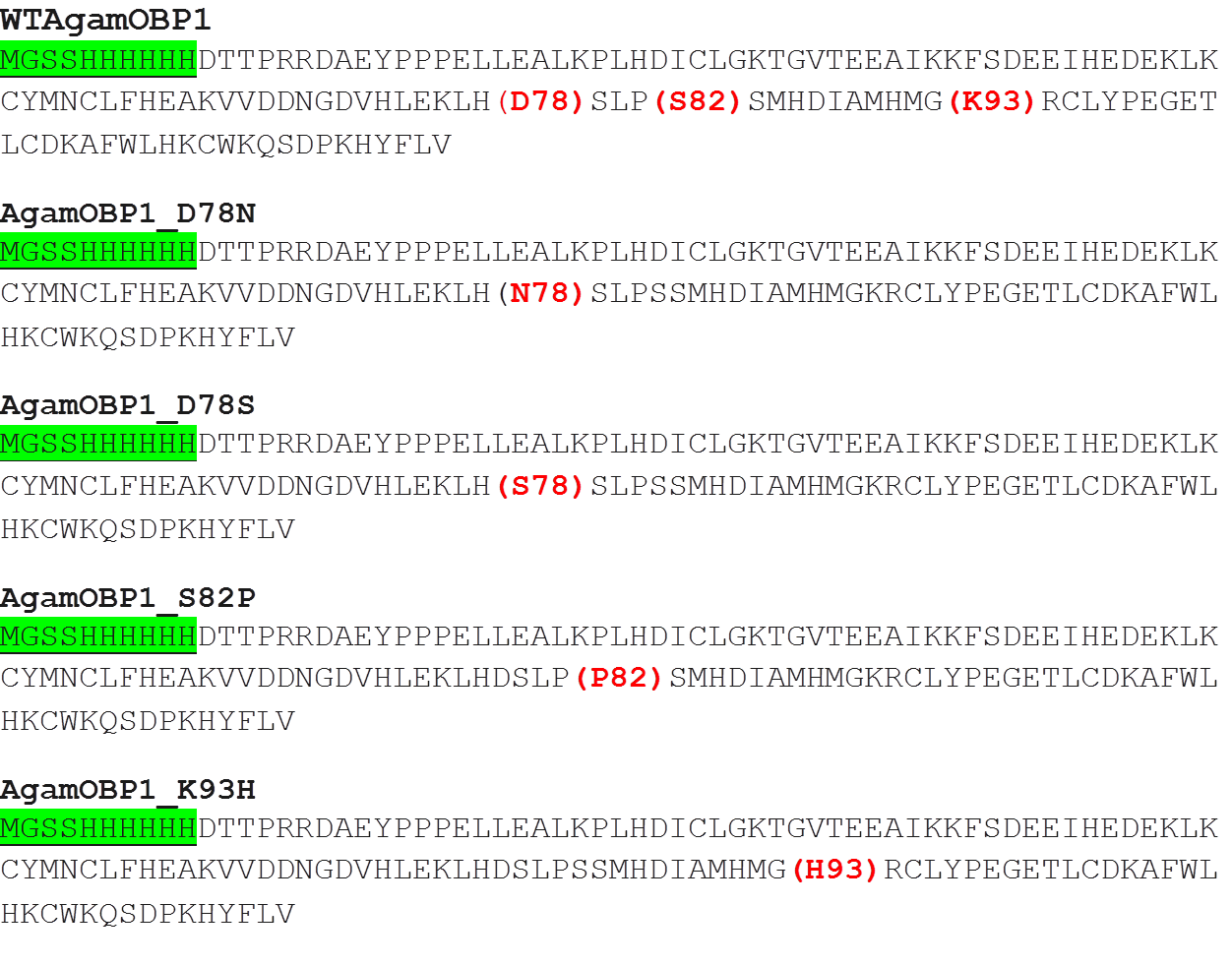


**Suppl. Material Figure S7a:** Amino acid sequences of 6-His tagged AgamOBP1 variants that were expressed and tested experimentally.

**
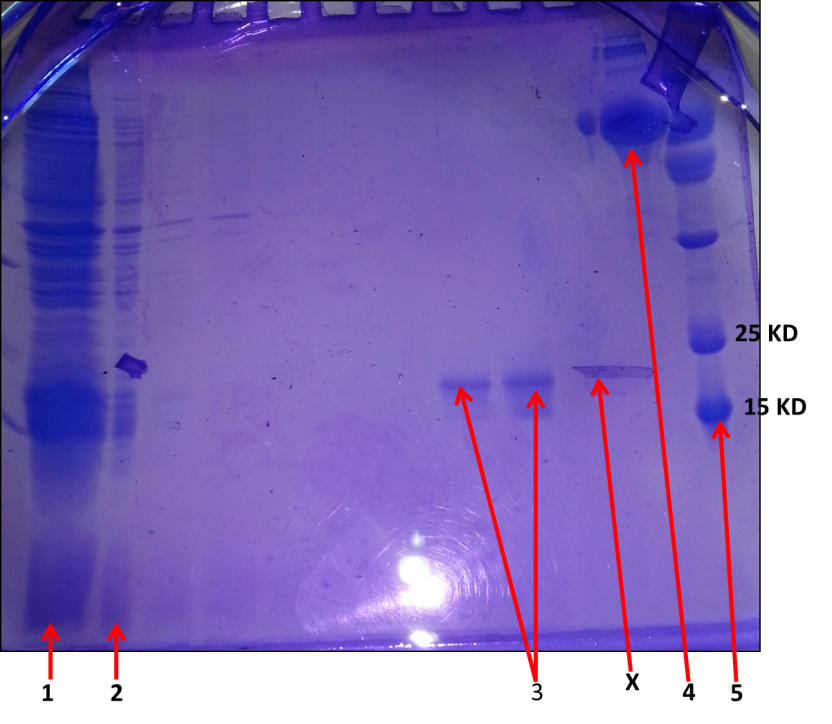
**

**Suppl. Material Figure S7b:** SDS –PAGE analysis of the purified samples of AgamOBP1 variants.

**All expressed proteins were verified for purity using SDS-PAGE.**  Here as an example a gel for AgamOBP1_ S82P is presented. The 6His-tagged protein was purified by affinity chromatography using a HisPrep FF 16/10 column as described in the material and methods section. In the gel; **(1)** = solubilised inclusion bodies pellet; **(2)** Extracted, urea/treated, dialysed sample (column input); **(3)** Elution fractions (5 ml each) containing purified AgamOBP1_ S82P (Expected MW: 15.72KD); **(4)** BSA standard (66.7 KD); **(5)** Molecular weight protein standards; and **(X)** an artefact – a piece of detached gel under the main gel.


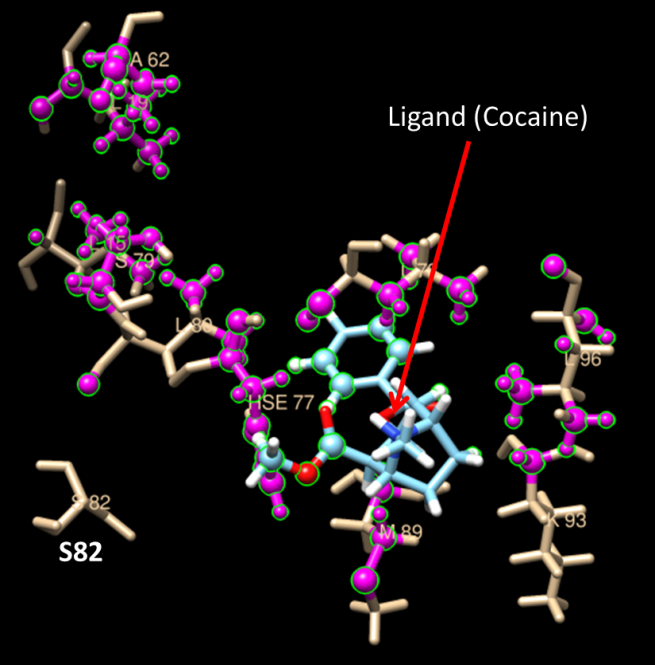

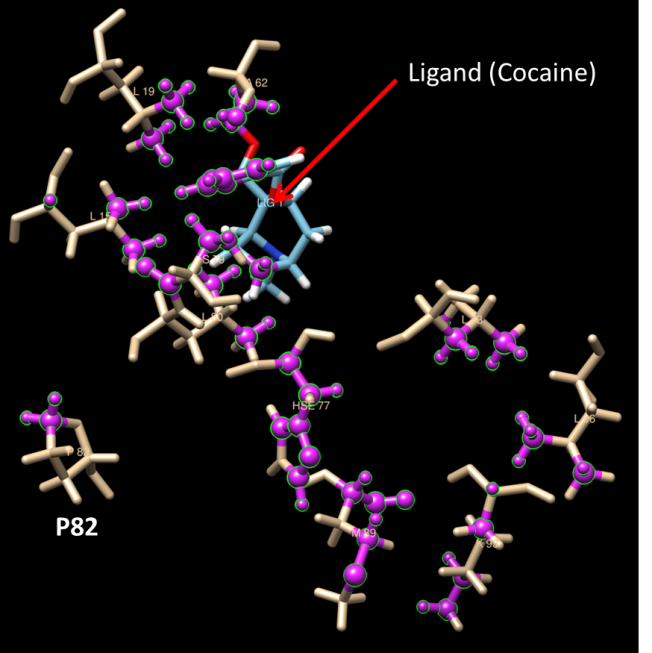


**(a)**

**(a)**


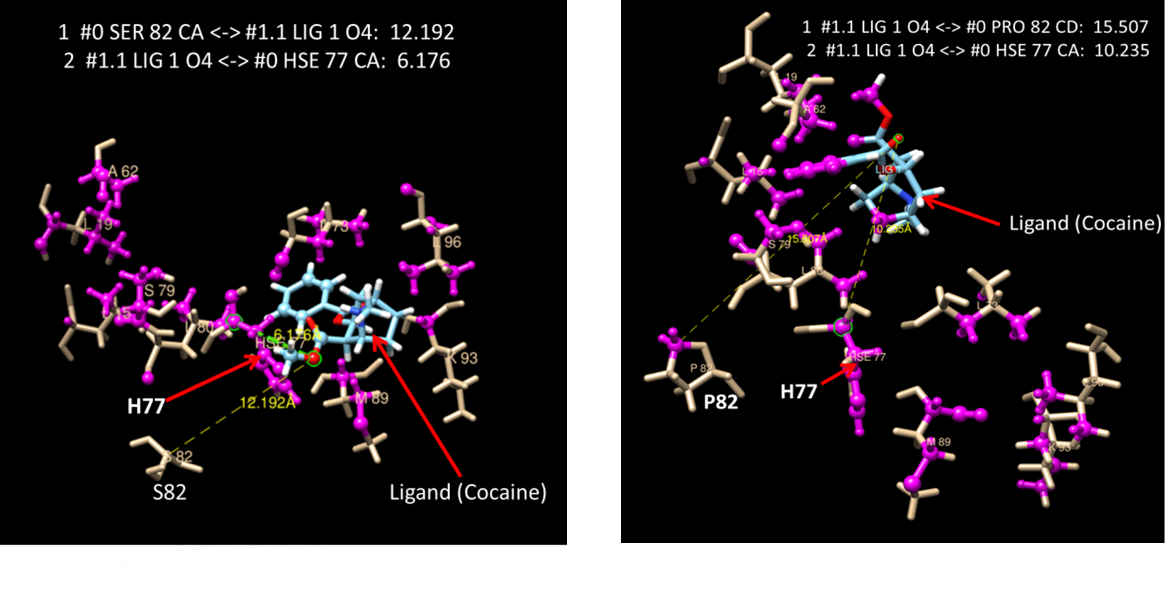


**(b)**

**(b)**

**Suppl. Material Figure S8:** **(a)** Structural analysis, contacts (**green**/pink Highlight) between cocaine ligand and mouth residues. **Left is** WTAgamOBP1 and **right is** AgamOBP1_S82P. The analysis was carried out using the Structural analysis/Find Clashes/Contacts tool of UCSF Chimera.

**(b)** Structural analysis, distance between cocaine ligand and mouth residues. **Left is WTAgamOBP1** and **right** is AgamOBP1_S82P. The analysis was carried out using the Structural analysis/Distances tool of UCSF Chimera. #0 = mouth residues in the model, while #1.1 = ligand number –there is only one ligand in this case, cocaine.


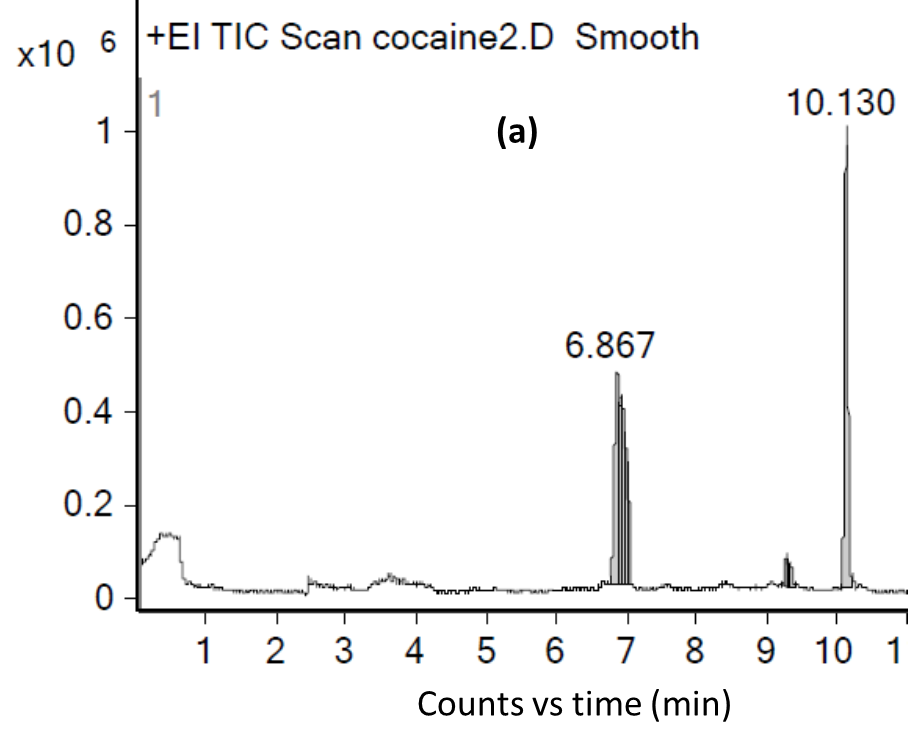


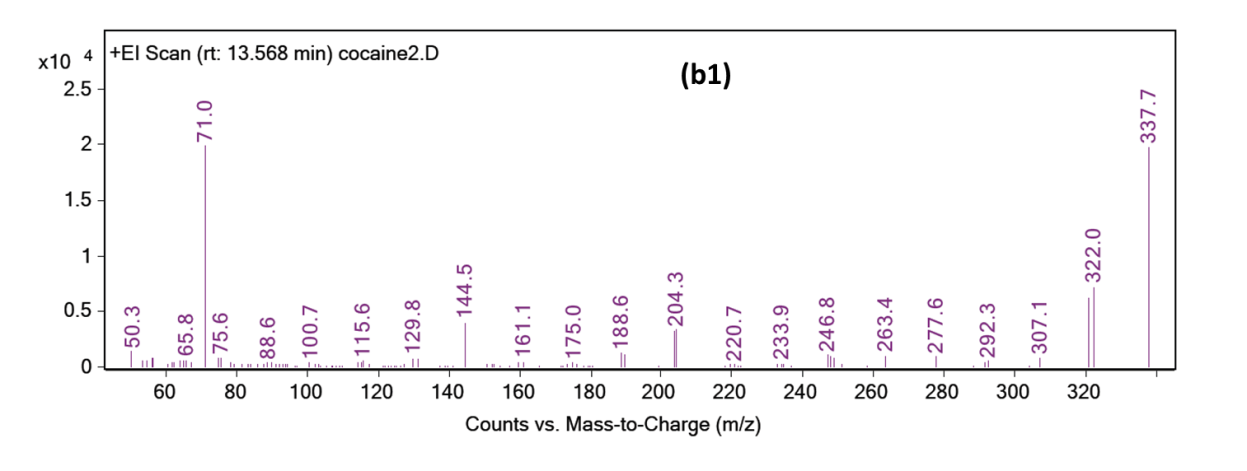


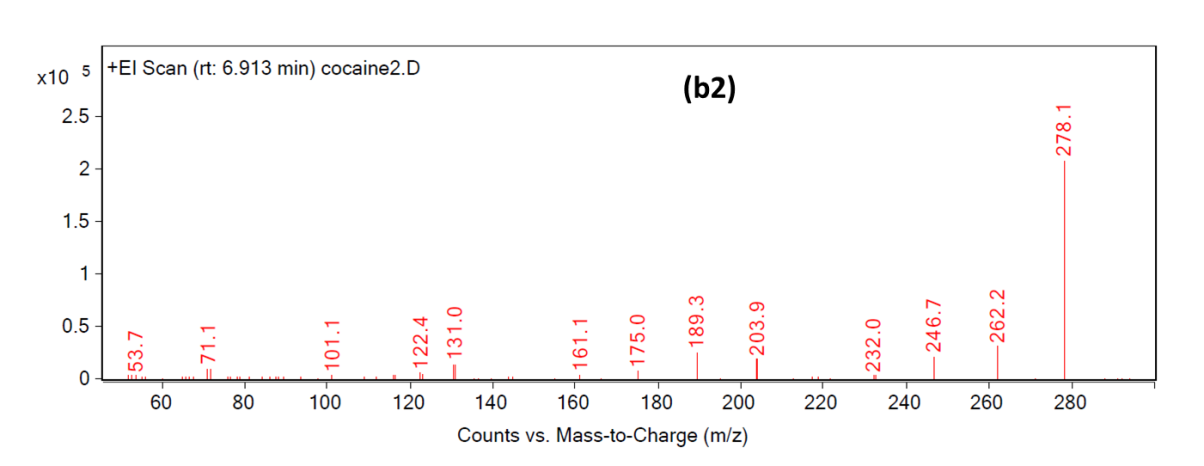


**Suppl. Material Figure S9: GC-MS analysis of Headspace of Cocaine-HCl sample. (a)** GC Chromatogram showing two major peaks, 10.13 minutes (cocaine HCl) (mass spectrum **b1, molecular ion 337.7**), 6.867 minutes (thermal decomposition product attributed to cocaine HCl minus COOCH_3_ (mass spectrum **b2, molecular ion 278.1**).

**Supplementary Material Table S1: Potential stable mutants around the active site of AgamOBP1.** These mutants were identified by analysis the X- ray structure of AgamOBP1 (PDB ID: 2ERB) using Prediction of Protein Mutants Stability Changes (PoPMuSiC) server.

**Supplementary Material Table S2:** For docking experiments, target drug molecules in addition to the fluorescence probe 1-NPN were investigated. The latter is used in displacement binding experiments to determine the affinities of binding of target ligands. The actual docking was done using the Swissdock server (Swiss Institute of Bioinformatics (http://swissdock.vital-it.ch/docking) which operates using “EADock DSS software”. Each of the 28 potential mutants for AgamOBP1 plus the WT was docked with each of the seven drug target ligands. During the docking procedure, the software generates many binding modes simultaneously and their energies are estimated. The binding modes with the most favourable energies were evaluated and clustered. For each docking outcome up to 42 clusters were generated, each with several rankings, a cluster is a binding mode and within it there are several ranks, each rank has slightly different energy value compared to another rank, some of those ranks are just repetitions, a much bigger energy differences occurs between one binding mode to the other. The data were visualised using the plug in “UCF Chimera”. Initially all non- binding pocket clusters were eliminated. Then the most energetic binding pocket cluster was selected and refined by eliminating any repeated ranks **(Suppl. Material Figure 3)** . Binding energy values were used to define mutant variants with potentially stronger binding affinity towards a given ligand as the lower the binding energy the stronger the binding and vice versa. The mean value for the binding energy for the drugs for each protein variant was calculated, and the difference between the mean value of a given mutant and the WT mean value was recorded. Any difference value that was ≤ -1.0 kcal/mol was used as an indicator of potentially stronger binding for that particular mutant than a WT across all of the ligands tested.

**Supplementary Material Table S3**: Summarising the dimensions of the AgamOBP1 mutant variants main binding pockets (active sites) and their respective mouths openings in comparison to the WT protein. The pockets were generated by analysing the structures of each of the protein using CASTp Server. In the Table: **(A)** = Area (Solvent accessible surface-AS) Å^2^; **(B)** = Area (Molecular surface-MS) Å^2^; **(C)** = Volume (Solvent accessible surface-AS) Å^3^; **(D)** = Volume (Molecular surface-MS)Å^3^; **(E)** = Pocket length (Å), **(F)** = Number of mouth openings; **(G)** = Mouth area (Solvent accessible surface-AS)Å^2^; **(H)** = Mouth area (Molecular surface-MS)Å^2^; **(I)** = Mouth Length (Solvent accessible surface-AS)Å; **(J)** = Mouth Length (Molecular surface-MS)Å . Highlighted variants were expressed and tested experimentally.

**Supplementary Material Table S4: Saturated Vapour Pressures of Drugs under investigation**
